# IL‑1 receptor antagonism attenuates renal fibrosis via RNF182‑driven MFN2 destabilization and mitochondrial dysfunction

**DOI:** 10.1038/s41420-025-02929-4

**Published:** 2025-12-29

**Authors:** Bo Yang, Qing Shao, Wei Wang, Maoting Li, Fanzhou Zeng, Xuezi Fu, Jun Liu, Cheng Xue, Nanmei Liu

**Affiliations:** 1 Department of Nephrology, Naval Medical Center of PLA, Naval Medical University, Shanghai, China; 2 Department of Rehabilitation Medicine, Changhai Hospital, Naval Medical University, Shanghai, China; 3 Department of dive medicine, Naval Medical Center of PLA, Naval Medical University, Shanghai, China; 4 Department of Nephrology, Shanghai Changzheng Hospital, Second Affiliated Hospital of Naval Medical University (SMMU), Shanghai, China

**Keywords:** Outcomes research, End-stage renal disease

## Abstract

Renal fibrosis is a major driver of chronic kidney disease (CKD) progression, yet targeted therapies remain limited due to incomplete understanding of key molecular mechanisms. While IL-1-mediated inflammation and mitochondrial dysfunction are recognized contributors, the precise links between IL-1 signaling, fibrosis, and mitochondrial homeostasis are unclear. Here, we investigated the therapeutic effects of recombinant human IL-1 receptor antagonist (rhIL-1Ra) in both acute (UUO) and chronic (5/6Nx) mouse models of kidney injury, as well as in vitro TGF-β1-stimulated kidney cells. rhIL-1Ra significantly attenuated renal fibrosis, inflammation, and functional impairment in vivo. Mechanistically, rhIL-1Ra suppressed TGF-β1-induced expression of the E3 ubiquitin ligase RNF182, which we show mediates MFN2 ubiquitination and degradation, leading to mitochondrial dysfunction. Inhibition of RNF182 by rhIL-1Ra stabilized MFN2, preserved mitochondrial respiration and ATP production, and reduced oxidative stress. Rescue experiments confirmed the centrality of the RNF182-MFN2 axis in fibrotic and mitochondrial injury. Our findings reveal a novel IL-1R/RNF182/MFN2 pathway linking inflammation to mitochondrial and fibrotic pathology, supporting RNF182 as a promising target and rhIL-1Ra as a potential therapy for CKD.

## Introduction

Chronic kidney disease (CKD) represents a growing global health burden, affecting millions worldwide and progressively leading to end-stage renal disease (ESRD), cardiovascular complications, and premature mortality [1, 2]. A hallmark pathological feature shared across nearly all etiologies of modern CKD is renal fibrosis, characterised by means of excessive accumulation of extracellular matrix (ECM) proteins, activation and proliferation of myofibroblasts, continual irritation, and eventual destruction of ordinary kidney structure [3, 4]. Despite advances in expertise its pathogenesis, cutting-edge therapeutic techniques, usually targeted on managing underlying conditions and controlling hypertension with renin-angiotensin system (RAS) inhibitors or, greater currently, SGLT2 inhibitors, best sluggish sickness development instead of halting or reversing fibrosis [5, 6]. Consequently, there remains an urgent unmet need for novel healing methods that directly goal the center mechanisms riding renal fibrosis [7].

Infection is intricately connected with the initiation and perpetuation of renal fibrosis [8]. Continual inflammatory responses create a pro-fibrotic microenvironment wealthy in cytokines and growth factors that drive pathological changes [9, 10]. Among these, the Interleukin-1 (IL-1) family, particularly IL-1β, performs a pivotal function thru its receptor IL-1R, contributing to tissue damage and inflammatory cell infiltration in each acute kidney injury (AKI) and CKD [11, 12]. Focused on the IL-1/IL-1R axis with sellers like recombinant human IL-1 receptor antagonist (rhIL-1Ra, Anakinra) thus gives a rational therapeutic method, evidently constructing upon the endogenous regulatory mechanism of IL-1Ra [13].

But, whilst the contribution of IL-1 signaling to renal inflammation is properly-set up, its precise effect on without delay orchestrating the fibrotic program within resident kidney cells, such as tubular epithelial cells and fibroblasts, remains less defined [14]. In particular, how IL-1R signaling intersects with canonical pro-fibrotic pathways, like the ones driven by means of TGF-β1, to modulate ECM production and myofibroblast activation at the molecular stage calls for further explanation beyond popular results [15]. Identifying the specific downstream mediators regulated by using IL-1R antagonism inside those intrinsic cellular fibrotic programs represents a vital understanding gap [16].

Crucial to kidney health and disease progression is the status of mitochondria [17]. These organelles are metabolic powerhouses essential for the excessive energy needs of renal cells, specially tubular epithelial cells worried in solute shipping [18]. Beyond organelles are metabolic powerhouses essential for the excessive energy needs of renal cells, specially tubular epithelial cells worried in solute shipping [19]. It is miles increasingly obtrusive that mitochondrial dysfunction—manifesting as impaired respiration capacity, excessive reactive oxygen species (ROS) production, altered dynamics, and defective mitophagy—is a essential pathogenic characteristic in both AKI and CKD, actively contributing to tubular damage, inflammation, and the improvement of interstitial fibrosis [20]. Therefore, strategies aimed at retaining mitochondrial integrity preserve sizeable healing promise [21].

Inside the complex machinery governing mitochondrial health, Mitofusin 2 (MFN2) stands out as a key regulatory protein located on the outer mitochondrial membrane [22]. MFN2 is essential for mediating mitochondrial fusion, a process critical for maintaining a healthy and interconnected mitochondrial network, permitting content mixing and functional complementation [23]. It also plays roles in mitochondrial transport, mitochondria-ER tethering, and mitophagy regulation. Emerging evidence suggests that MFN2 expression or function can be compromised in kidney disease models, and its restoration may confer protective effects [24]. However, the precise mechanisms governing MFN2 protein stability, particularly how it is regulated post-translationally in response to the combined inflammatory and fibrotic milieu characteristic of CKD, remain largely unexplored.

Post-translational modifications, specifically ubiquitination mediated by E3 ubiquitin ligases, provide a rapid and precise mechanism for controlling protein stability, localization, and activity, thereby shaping cellular responses [25]. The ubiquitin-proteasome system is increasingly implicated in renal pathophysiology [26]. Ring Finger Protein 182 (RNF182) is one such E3 ubiquitin ligase, whose functions have been investigated in other contexts, such as neuronal development and cancer [27]. but, its potential role within the kidney, specially whether it acts as a important regulatory node linking upstream signaling pathways (inclusive of the ones activated by IL-1 or TGF-β1) to the degradation of unique downstream goals involved in fibrosis or mitochondrial health, has now not been investigated [28]. Identifying this type of link could reveal novel therapeutic targets [29].

Through elucidating a unique signaling axis involving IL-1R/RNF182/MFN2 that governs mitochondrial health and fibrotic responses in kidney cells, this studies provides fullsize new insights into the pathogenesis of renal fibrosis. The identity of RNF182 as a capability healing target and the validation of rhIL-1Ra’s efficacy via this pathway underscore its potential as a centered healing method. these findings not simplest enhance our essential information but additionally provide a strong preclinical intent for exploring rhIL-1Ra for the remedy of CKD, doubtlessly supplying blessings by using addressing interconnected inflammatory, fibrotic, and mito-metabolic pathologies.

## Result

To evaluate the therapeutic efficacy of rhIL--1Ra in mitigating kidney fibrosis, we employed both an acute unilateral ureteral obstruction (UUO) model and a chronic 5/6 nephrectomy (5/6Nx) model of renal injury. Throughout the in vivo experiments presented, the experimental groups are consistently defined as follows: (1) Control: Healthy, untreated mice serving as a naive physiological baseline; (2) Sham: Mice that underwent sham surgical procedures corresponding to either the UUO or 5/6Nx model, serving as the rigorous surgical controls; (3) UUO / 5/6Nx: Mice subjected to the respective injury model (ureteral ligation or 5/6 nephrectomy) and treated with a vehicle, representing the disease state. (4) Treatment Groups (e.g., UUO + rhIL-1Ra, 5/6Nx + rhIL-1Ra): Mice subjected to the injury model and subsequently treated with rhIL-1Ra or other indicated therapeutic agents.

### rhIL-1Ra ameliorates renal dysfunction, inflammation, and fibrosis in mice subjected to unilateral ureteral obstruction

**Fig. 1:**
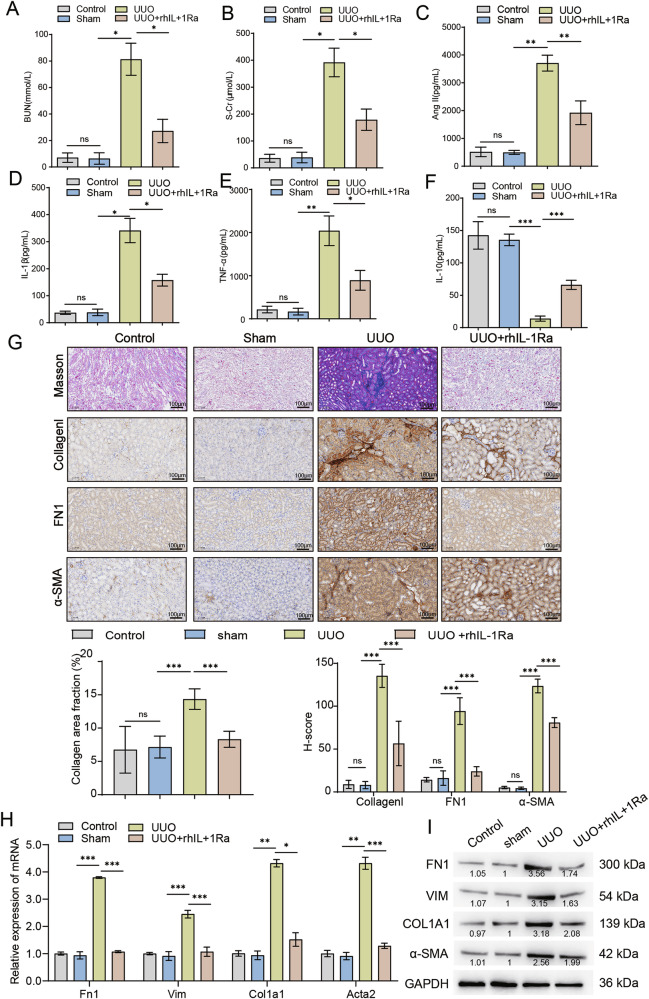
Therapeutic administration of rhIL-1Ra attenuates renal dysfunction, systemic inflammatory markers, and fibrotic changes in the murine Unilateral Ureteral Obstruction (UUO) model. Quantification of serum levels for BUN (**A**) and S-Cr (**B**) as indicators of renal function. Serum concentrations of Ang II (**C**), IL-1β (**D**), TNF-α (**E**), and IL-10 (**F**), determined by ELISA. **G** Representative histological (Masson’s Trichrome) and immunohistochemical (IHC) images of kidney sections (top panels), with corresponding quantitative analysis shown below (bottom panels). Quantification includes collagen area fraction for Masson’s staining and H-score analysis for Collagen I, FN1, and α-SMA IHC. Scale bars = 100 μm. **H** Relative mRNA expression levels of key fibrotic genes *(Fn1*, *Vim*, *Col1a1*, *Acta2* in kidney tissue lysates, measured by qRT-PCR). Data were normalized to Gapdh expression and are shown relative to the Sham group. **I** Representative Western blot analysis displaying the protein levels of FN1, VIM, COL1A1, and α-SMA in kidney tissue lysates from Control, Sham, UUO, and UUO+rhIL-1Ra groups. Blots are representative of three independent experiments, and the values below the bands indicate the mean relative densitometric intensity (*n* = 3) normalized to GAPDH. Approximate molecular weight markers (kDa) are indicated on the right. Data are presented as mean ± SEM (*n* = 6 mice / group). Statistical significance between groups was assessed using One-way ANOVA followed by Tukey’s multiple comparison post hoc test. *p < 0.05, **p < 0.01, ***p < 0.001.

To investigate the therapeutic potential of recombinant human rhIL-1Ra on kidney fibrosis in vivo, we utilized the UUO mouse model. We assessed renal function by measuring serum levels of BUN and S-Cr. Compared to the Control and Sham groups, mice subjected to UUO exhibited significantly elevated levels of BUN (Fig. 1A) and S-Cr (Fig. 1B), indicating impaired renal function. Treatment with rhIL-1Ra significantly attenuated these increases, suggesting a protective effect on kidney function. Given the role of inflammation and Ang-II in the pathogenesis of kidney fibrosis, we measured their levels in serum. UUO surgery led to a significant increase in serum Ang-II levels compared to the Sham group, which was significantly reduced by rhIL-1Ra administration (Fig. 1C). Furthermore, UUO resulted in a marked elevation of pro-inflammatory cytokines, including IL-1β (Fig. 1D) and TNF-α (Fig. 1E), compared to Sham controls. Conversely, the level of the anti-inflammatory cytokine IL-10 was significantly decreased in the UUO group (Fig. 1F). Treatment with rhIL-1Ra effectively suppressed the UUO-induced increase in IL-1β and TNF-α (Figs. 1D, 1E) and partially restored the levels of IL-10 (Fig. 1F), indicating that rhIL-1Ra alleviates the inflammatory response associated with UUO.To at once compare the impact of rhIL-1Ra on renal fibrosis development, we accomplished histological analyses on kidney tissues. Masson’s trichrome staining revealed extensive collagen deposition (indicated via blue staining) inside the kidneys of UUO mice, characteristic of tremendous fibrosis. This collagen accumulation became markedly reduced in the UUO+rhIL-1Ra group (Fig. 1G, pinnacle row). IHC staining similarly supported these findings. The expression of key ECM proteins, Collagen I (Fig. 1G, second row) and FN1 (Fig. 1G, third row), changed into considerably accelerated in the renal interstitium of UUO mice compared to Sham controls. Treatment with rhIL-1Ra notably diminished the staining intensity for each Collagen I and FN1. additionally, IHC staining for α-SMA, a marker of myofibroblast activation (a key cell type driving fibrosis), showed a tremendous growth in the UUO group, which turned into additionally significantly reduced upon rhIL-1Ra treatment (Fig. 1G, bottom row). steady with the histological observations, we examined the expression of fibrosis-related genes at the mRNA level using qRT-PCR. The relative mRNA expression levels of Fn1, Vimentin, Col1a1, and α-sma were all extensively upregulated inside the kidney tissues of UUO mice as compared to the Sham group. Treatment of rhIL-1Ra appreciably suppressed the UUO-induced upregulation of these fibrotic marker genes (Fig. 1H). ultimately, we showed those findings at the protein level using Western blotting. Protein expression of FN1, Vimentin, COL1A1, and α-SMA became significantly higher in kidney lysates from UUO mice compared to the Sham group. treatment with rhIL-1Ra markedly reduced the protein levels of those fibrotic markers (Fig. 1I). collectively, those consequences reveal that rhIL-1Ra treatment efficaciously ameliorates renal dysfunction, reduces inflammation, and attenuates the improvement of renal fibrosis in the UUO mouse model.

### IL-1Ra alleviates renal injury, inflammation, and fibrosis in a 5/6 nephrectomy model of chronic kidney disease

**Fig. 2:**
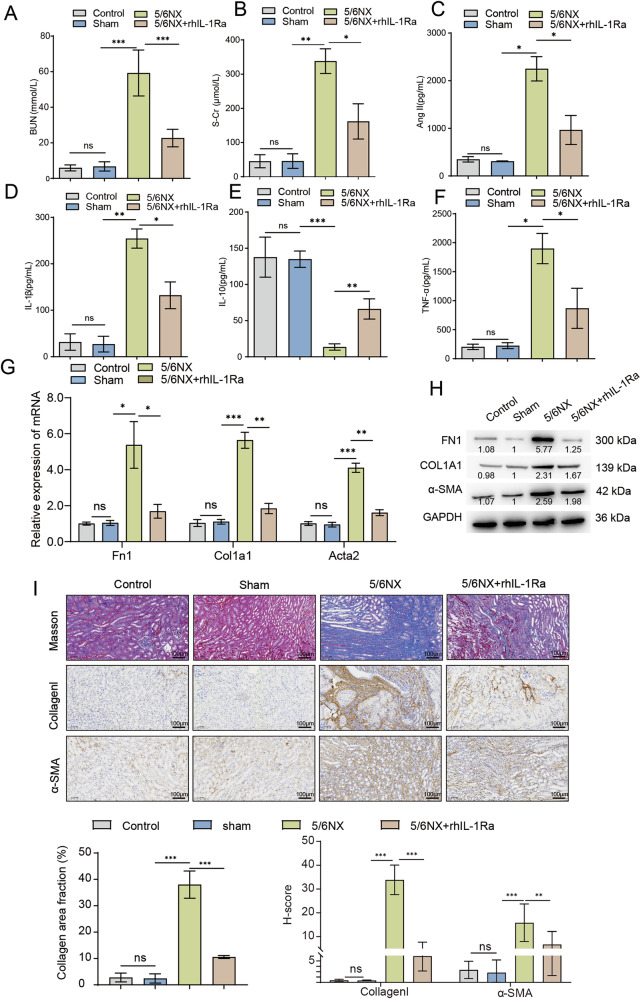
rhIL-1Ra administration improves renal function and attenuates systemic inflammation markers and renal fibrosis in the murine 5/6 nephrectomy (5/6Nx) model of chronic kidney disease. Serum levels of BUN (**A**) and S-Cr (**B**), indicators of renal function. Serum concentrations of Ang II (**C**), IL-1β (**D**), IL-10 (**E**), and TNF-α (**F**), measured by ELISA. **G** Relative mRNA abundance of fibrotic marker genes (*Fn1*, *Col1a1*, *Acta2* [encoding α-SMA]) in kidney tissue lysates, determined by qRT-PCR. Expression was normalized to Gapdh and is presented relative to the Sham group. **H** Representative Western blot analysis showing protein levels of FN1, COL1A1, and α-SMA in kidney tissue lysates. Blots are representative of three independent experiments, and the values below the bands indicate the mean relative densitometric intensity (*n* = 3) normalized to GAPDH. Approximate molecular weights (kDa) are indicated. **I** Representative photomicrographs of kidney sections. Top row: Masson’s Trichrome staining showing collagen deposition (blue). Middle row: IHC staining for Collagen I (brown). Bottom row: IHC staining for α-SMA (brown), with corresponding quantitative analysis shown below (bottom panels).Scale bars = 100 μm. Data are presented as mean ± SEM (*n* = 6 mice / group. Statistical significance between groups was determined using One-way ANOVA followed by Tukey’s multiple comparison post hoc test. *p < 0.05, **p < 0.01, ***p < 0.001.

To further evaluate the therapeutic efficacy of rhIL-1Ra in a CKD-relevant setting, we employed the 5/6Nx mouse model. Assessment of renal function revealed that 5/6Nx mice exhibited significantly elevated serum BUN (Fig. 2A) and S-Cr (Fig. 2B) levels compared to sham-operated controls, confirming the successful establishment of renal insufficiency. Administration of rhIL-1Ra markedly attenuated these increases, indicating an improvement in renal function. We next measured serum Ang-II and key inflammatory cytokines. Ang-II was significantly increased in the 5/6Nx group compared to the Sham group, and this elevation was notably reduced following rhIL-1Ra treatment (Fig. 2C). Similarly, serum IL-1β (Fig. 2D) and TNF-α (Fig. 2F) levels were markedly elevated in 5/6Nx mice, while IL-10 (Fig. 2E) was significantly decreased. rhIL-1Ra treatment effectively lowered IL-1β and TNF-α levels and partially restored IL-10 (Fig. 2D–F), reflecting modulation of the systemic inflammatory response. To investigate the impact of rhIL-1Ra on renal fibrosis, we analyzed the expression of fibrotic markers at the mRNA level. qRT-PCR of kidney tissue showed significant upregulation of Fn1, Col1a1, and Acta2 mRNA in 5/6Nx mice relative to the Sham group, which was significantly suppressed by rhIL-1Ra (Fig. 2G). In agreement with these findings, Western blot analysis demonstrated increased protein expression of FN1, COL1A1, and α-SMA in 5/6Nx kidneys, which was markedly reduced by rhIL-1Ra treatment (Fig. 2H). Histological analysis further supported these results. Masson’s trichrome staining revealed substantial collagen deposition in the renal interstitium of 5/6Nx mice, which was markedly reduced following rhIL-1Ra administration (Fig. 2I, top row). Immunohistochemistry showed increased collagen I (Fig. 2I, middle row) and α-SMA (Fig. 2I, bottom row) expression in 5/6Nx kidneys, both of which were attenuated by rhIL-1Ra.Collectively, these results demonstrate that rhIL-1Ra treatment ameliorates renal dysfunction, suppresses inflammation, and attenuates renal fibrosis in the 5/6Nx mouse model of CKD.

### rhIL‑1Ra attenuates TGF‑β1–induced pro‑fibrotic and pro‑inflammatory responses in kidney epithelial and fibroblast cell lines

Because rhIL‑1Ra mitigated renal fibrosis in vivo (Figs. 1, 2), we examined whether it acts directly on kidney parenchymal and stromal cells. Human proximal tubular HK‑2 and rat renal fibroblast NRK‑49 F cells both expressed IL‑1 receptor (Fig. S1A).

To establish the effective concentration of rhIL-1Ra and confirm its mechanism, we performed comprehensive dose-response analyses. We found that rhIL-1Ra potently inhibited the TGF-β1-induced fibrotic program in a clear dose-dependent manner. This effect was evident at the mRNA level across both HK-2 and NRK-49F cells (Fig. S1B) and was subsequently confirmed at the protein level for FN1, COL1A1, Vimentin, and α-SMA in HK-2 and NRK-49F cells (Fig. S1C).

**Fig. 3:**
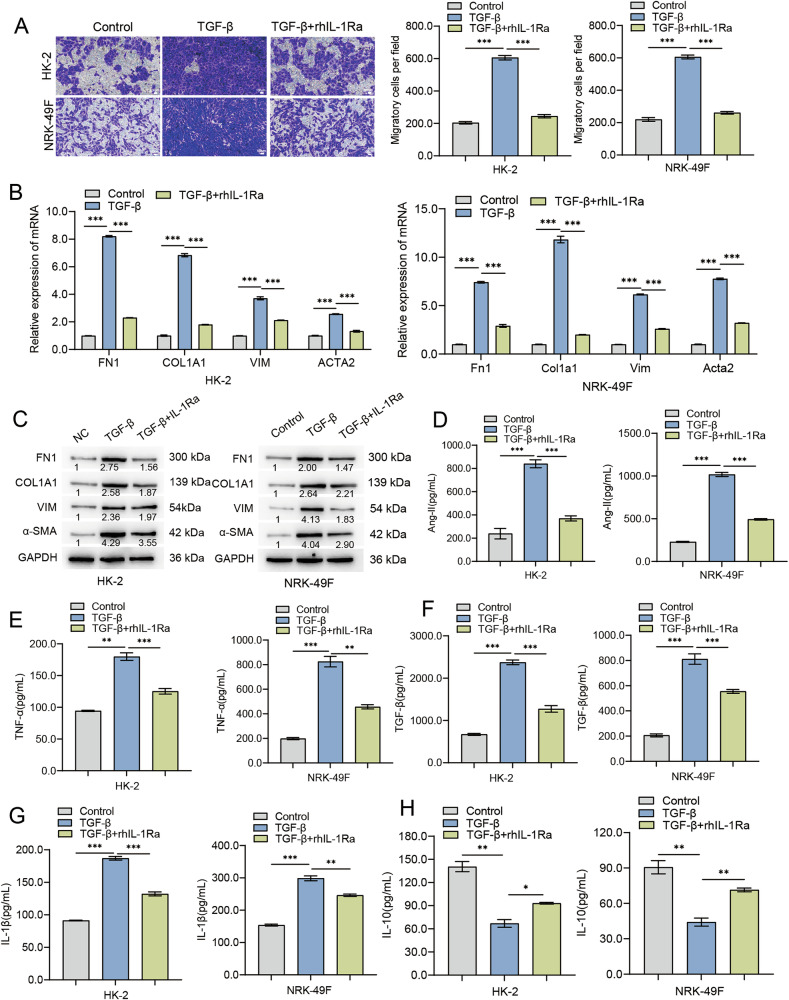
rhIL-1Ra directly counteracts TGF-β1-induced pro-fibrotic responses and modulates secreted factors in HK-2 and NRK-49F. **A** Cell migration assessment using Transwell assays. Left panels show representative images of crystal violet-stained migrated HK-2 (top) and NRK-49F (bottom) cells. Right panels show quantification of migratory cells per field. Scale bars = 100 μm. **B** Relative mRNA expression levels of fibrotic marker genes (*FN1/Fn1*, *COL1A1/Col1a1*, *VIM/Vim*, *ACTA2/Acta2* [encoding α-SMA]) in HK-2 (left) and NRK-49F (right) cells, determined by qRT-PCR. Data were normalized to GAPDH/Gapdh expression and are shown relative to the Control group. **C** Representative Western blot analysis showing protein levels of FN1, COL1A1, VIM, and α-SMA in HK-2 (left) and NRK-49F (right) cell lysates. Blots are representative of three independent experiments, and the values below the bands indicate the mean relative densitometric intensity (*n* = 3) normalized to GAPDH. Approximate molecular weight markers (kDa) are indicated. Concentrations of secreted factors in cell culture supernatants, measured by ELISA: Ang-II (**D**), TNF-α (**E**), TGF-β (**F**), IL-1β (**G**), and IL-10 (**H**) from HK-2 (left graph in each panel) and NRK-49F (right graph in each panel) cells. Data in bar graphs (**A**, **B**, **D**–**H**) are presented as mean ± SEM. Statistical significance was determined using One-way ANOVA followed by Tukey’s multiple comparison post-hoc test. *p < 0.05, **p < 0.01, ***p < 0.001.

Based on these robust dose-response data, we selected a concentration of 100 ng/mL, which produced a strong and near-maximal inhibitory effect. Cells were exposed to TGF‑β1 (10 ng/mL, 24 h) with or without rhIL‑1Ra (100 ng/mL; pre‑incubation 1 h). TGF‑β1 markedly increased Transwell migration in both lines; rhIL‑1Ra significantly suppressed this response (Fig. 3A) and was further confirmed by an accelerated rate of wound closure in a complementary wound healing assay (Fig. S1D). Crucially, rhIL-1Ra co-treatment significantly suppressed both forms of TGF-β1-induced cell migration.

TGF‑β1 robustly induced transcripts encoding fibronectin (*FN1/Fn1*), type I collagen (*COL1A1/Col1a1*), vimentin (*VIM/Vim*), and α‑smooth muscle actin (*ACTA2/Acta2*); rhIL‑1Ra significantly blunted these inductions in HK‑2 and NRK‑49 F cells (Fig. 3B). Immunoblotting confirmed parallel reductions in FN1, COL1A1, VIM, and α‑SMA proteins (Fig. 3C).

Analysis of conditioned media revealed that TGF‑β1 stimulation increased Ang II and TNF‑α, and elevated IL‑1β while reducing IL‑10; co‑treatment with rhIL‑1Ra significantly attenuated each of these changes in both cell types (Fig. 3D, E, G, H). Measured TGF‑β1 concentrations in the medium were, as expected, higher after TGF‑β1 treatment (reflecting residual exogenous ligand plus cell‑derived production); recoverable levels were reduced by rhIL‑1Ra (Fig. 3F).

These data indicate that IL‑1 R antagonism directly counters multiple TGF‑β1–driven pro‑fibrotic and pro‑inflammatory programs in kidney epithelial and fibroblast models, providing a mechanistic bridge to the in vivo protective effects of rhIL‑1Ra.

### RNF182 mediates the anti-fibrotic effects of rhIL-1Ra in vitro

**Fig. 4:**
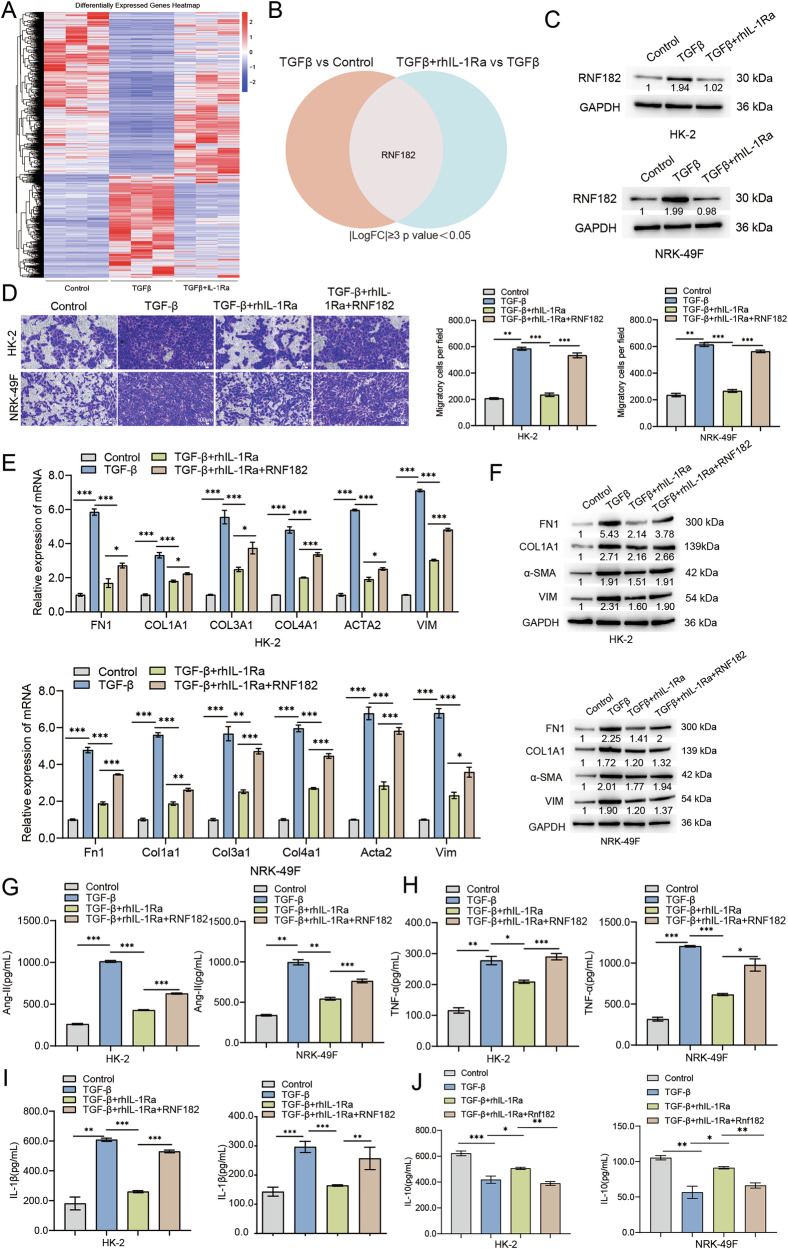
RNF182 expression is regulated by TGF-β1 and rhIL-1Ra, and RNF182 overexpression reverses the anti-fibrotic effects of rhIL-1Ra in vitro. **A** Heatmap displaying hierarchical clustering of differentially expressed genes identified by RNA-seq in HK-2 cells following treatment with Control, TGF-β1, or TGF-β1+rhIL-1Ra (*n* = 3 /group). Color scale indicates Z-score normalized expression values (Red: higher expression, Blue: lower expression). **B** Venn diagram illustrating the overlap between genes significantly upregulated by TGF-β1 (vs Control) and genes significantly downregulated by TGF-β1+rhIL-1Ra (vs TGF-β1 alone) in the RNA-seq dataset. Cutoffs: |Log2 FoldChange | ≥ 3 and adjusted *p* value < 0.05. RNF182 is highlighted as a gene meeting these criteria. **C** Representative Western blot analysis confirming RNF182 protein levels in HK-2 (top) and NRK-49F (bottom) cells treated with Control, TGF-β1, or TGF-β1+rhIL-1Ra. Blots are representative of three independent experiments, and the values below the bands indicate the mean relative densitometric intensity (*n* = 3) normalized to GAPDH. **D** Assessment of cell migration using Transwell assays in HK-2 (top) and NRK-49F (bottom) cells under control, TGF-β1, TGF-β1+rhIL-1Ra, and TGF-β1+rhIL-1Ra + RNF182 overexpression conditions. Left panels: representative images of migrated cells stained with crystal violet. Right panels: quantification of migratory cells per field. Scale bars = 100 μm. **E** Relative mRNA expression levels of fibrotic marker genes (*FN1/Fn1*, *COL1A1/Col1a1*, *COL3A1/Col3a1*, *COL4A1/Col4a1*, *ACTA2/Acta2* [encoding α-SMA], *VIM/Vim*) in HK-2 (top) and NRK-49F (bottom) cells under the indicated treatment and RNF182 overexpression conditions, measured by qRT-PCR. Data normalized to GAPDH/Gapdh and shown relative to the corresponding Control group. **F** Representative Western blot analysis showing protein levels of fibrotic markers (FN1, COL1A1, VIM, α-SMA) in HK-2 (top) and NRK-49F (bottom) cells under the indicated treatment and RNF182 overexpression conditions. Blots are representative of three independent experiments, and the values below the bands indicate the mean relative densitometric intensity (*n* = 3) normalized to GAPDH. Approximate molecular weights (kDa) are indicated. Concentrations of secreted factors in cell culture supernatants, measured by ELISA: Ang-II (**G**), TNF-α (**H**), IL-1β (**I**), and IL-10 (**J**) from HK-2 (left graph in each panel) and NRK-49F (right graph in each panel) cells under the indicated treatment and RNF182 overexpression conditions. Data are presented as mean ± SEM. Statistical significance was determined using One-way ANOVA followed by Tukey’s multiple comparison post-hoc test. *p < 0.05, **p < 0.01, ***p < 0.001.

To elucidate the downstream mechanisms underlying the therapeutic effects of rhIL-1Ra observed in vivo and in vitro, we performed RNA-seq analysis on HK-2 cells treated with vehicle (control), TGF-β1, or TGF-β1+ rhIL-1Ra. Hierarchical clustering revealed distinct gene expression profiles among the three groups, with numerous genes differentially regulated by TGF-β1 and subsequently modulated by rhIL-1Ra treatment (Fig. 4A). We focused on identifying potential mediators that were significantly upregulated by TGF-β1 compared to control and notably downregulated by rhIL-1Ra co-treatment relative to TGF-β1 alone (criteria: |Log2FoldChange | ≥3 and *p* < 0.05). Venn diagram analysis highlighted Ring Finger protein RNF182 as a prominent candidate meeting these criteria (Fig. 4B). qRT-PCR validation confirmed that TGF-β1 markedly increased transcript levels of CDH3, RNF182, and GABRA5, in both HK-2 and NRK-49F cells. In accordance with our screening criteria, co-treatment with rhIL-1Ra significantly attenuated the TGF-β1-induced upregulation of RNF182 and LRRC32, while the effects on GABRA5 were variable and did not reach statistical significance under the same conditions (Fig. S2A). RNF182 was prioritized for subsequent analyses owing to its mechanistic relevance to ubiquitin-dependent protein regulation and mitochondrial homeostasis. To further validate RNF182 at the protein level, we performed Western blot analysis. Consistent with the RNA-seq and qRT-PCR data, TGF-β1 stimulation significantly elevated RNF182 protein expression in both HK-2 and NRK-49F cells, and this increase was notably attenuated by rhIL-1Ra co-treatment (Fig. 4C). To definitively dissect the upstream signaling crosstalk governing RNF182 expression, we next investigated the necessity of the proposed IL-1R-dependent autocrine loop. We utilized a validated shRNA approach to effectively silence IL-1R expression (knockdown efficiency shown in Fig. S2B, C). Strikingly, in cells with IL-1R knockdown, the ability of TGF-β1 to induce RNF182 was almost completely abolished, an effect observed at both the mRNA and protein levels (Fig. S2D, E). This profound suppression was mirrored by a significant blunting of the entire downstream pro-fibrotic program, including the expression of FN1, COL1A1, and other markers (Fig. S2F, G). These results provide direct causal evidence that the IL-1R signaling axis is indispensable for mediating the TGF-β1-driven induction of RNF182 and the subsequent fibrotic response.

To investigate whether RNF182 plays a causal role in mediating the effects of rhIL-1Ra, we conducted rescue experiments by overexpressing RNF182 in cells treated with TGF-β1 and rhIL-1Ra. We first confirmed successful RNF182 overexpression, as transfection with the RNF182 expression vector led to a robust increase in RNF182 mRNA and protein levels compared to negative control (NC) vector in both HK-2 and NRK-49F cells (Fig. S2H, I). Functionally, RNF182 overexpression significantly reversed the inhibitory effect of rhIL-1Ra on TGF-β1-induced cell migration in both HK-2 and NRK-49F cells (Fig. 4D). We next examined whether RNF182 overexpression could counteract the suppressive effects of rhIL-1Ra on fibrotic marker expression. qRT-PCR showed that RNF182 overexpression largely abrogated the ability of rhIL-1Ra to reduce TGF-β1-induced mRNA levels of Fn1, Col1a1, Col3a1, Col4a1, α-SMA, and Vim in both cell lines (Fig. 4E). Likewise, Western blot analysis demonstrated that RNF182 overexpression partially reversed the reductions in FN1, COL1A1, α-SMA, and VIM protein levels achieved by rhIL-1Ra in TGF-β1-stimulated cells (Fig. 4F).We further evaluated whether RNF182 mediates the effects of rhIL-1Ra on secreted factors. Overexpression of RNF182 markedly reversed the inhibitory effects of rhIL-1Ra on TGF-β1-induced secretion of Ang-II (Fig. 4G) and TNF-α (Fig. 4H) in both cell types. Additionally, RNF182 overexpression significantly counteracted the suppressive effect of rhIL-1Ra on IL-1β secretion (Fig. 4I). Conversely, the ability of rhIL-1Ra to partially restore IL-10 levels in TGF-β1-treated cells was substantially diminished upon RNF182 overexpression (Fig. 4J). Taken together, these results strongly suggest that RNF182 is upregulated during TGF-β1-induced fibrotic responses, and that the therapeutic, anti-fibrotic effects of rhIL-1Ra are, at least in part, mediated through downregulation of RNF182.

### rhIL-1Ra regulates the RNF182-mediated ubiquitination and stability of MFN2

**Fig. 5:**
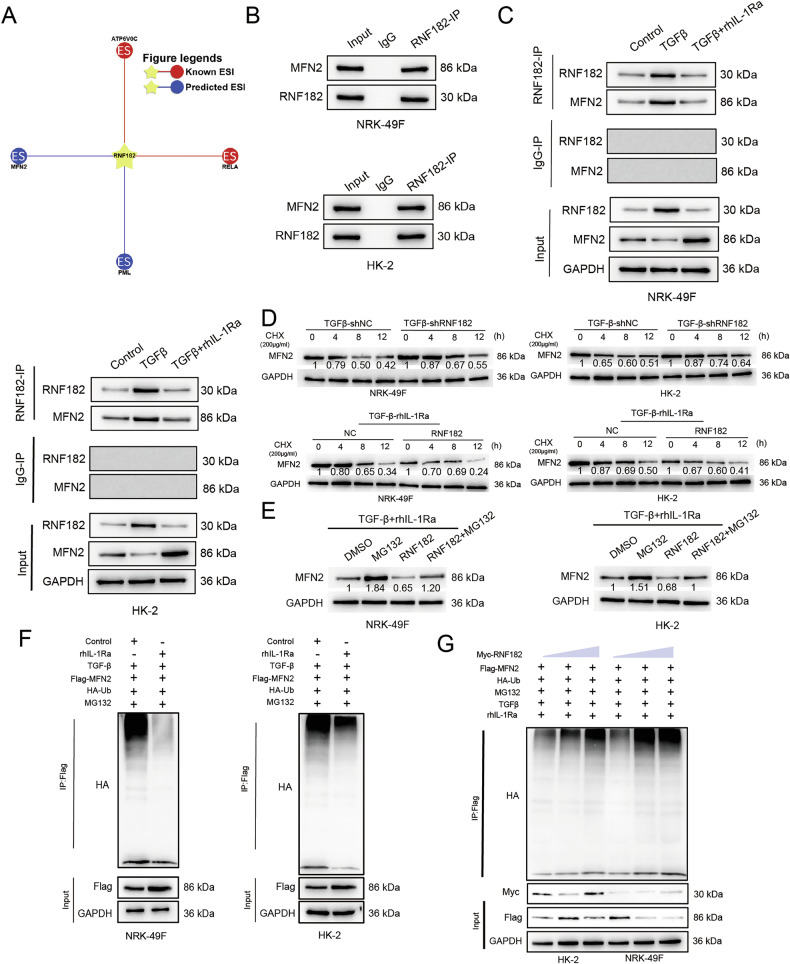
RNF182 interacts with, ubiquitinates, and promotes the degradation of MFN2, a process modulated by TGF-β1 and rhIL-1Ra. **A** Bioinformatic network prediction illustrating potential E3 substrate interactions (ESI) for RNF182, highlighting MFN2 as a predicted interactor based on UbiBrowser(http://ubibrowser.bio-it.cn/ubibrowser_v3/). **B** Co-IP demonstrating endogenous interaction between RNF182 and MFN2. Lysates from NRK-49F (top) and HK-2 (bottom) cells were subjected to IP with anti-RNF182, or control IgG antibodies, followed by Western blot for the indicated proteins. Input lanes show protein levels before IP. **C** Co-IP analysis showing the effect of treatments on the endogenous RNF182-MFN2 interaction. NRK-49F (top) and HK-2 (bottom) cells were treated with vehicle (Control), TGF-β1, or TGF-β1+rhIL-1Ra. Lysates were immunoprecipitated with anti-RNF182 or control IgG, followed by WB for RNF182 and MFN2. Input lanes are shown. **D** CHX chase assays assessing MFN2 protein stability. Top panels: NRK-49F and HK-2 cells treated with TGF-β1 were transduced with control shRNA (shNC) or RNF182 shRNA (shRNF182). Bottom panels: NRK-49F and HK-2 cells treated with TGF-β1+rhIL-1Ra were transfected with control vector (NC) or an RNF182 overexpression vector. CHX (200 μg/mL) was added (time 0), and cells were harvested at the indicated time points (h). MFN2 and GAPDH protein levels were analyzed by Western blot analysis. **E** Effect of proteasome inhibition on RNF182-mediated MFN2 levels. NRK-49F (left) and HK-2 (right) cells were treated with TGF-β1+rhIL-1Ra, transfected with control or RNF182 vector, and then treated with DMSO (vehicle) or the proteasome inhibitor MG132 (10 μM) for 6 h. MFN2 and GAPDH levels were assessed by Western blot analysis. **F** In vivo ubiquitination assay assessing the effect of TGF-β1 and rhIL-1Ra. HK-2 (left) and NRK-49F (right) cells were co-transfected with plasmids encoding Flag-tagged MFN2 (Flag-MFN2) and HA-tagged Ubiquitin (HA-Ub). Cells were treated with vehicle (Control), TGF-β1, or TGF-β1+rhIL-1Ra, followed by MG132 treatment before lysis under denaturing conditions. Flag-MFN2 was immunoprecipitated (IP: Flag), and ubiquitination was detected by Western blot analysis using an anti-HA antibody. Input lysates were blotted for Flag-MFN2 and GAPDH. **G** In vivo ubiquitination assay demonstrating RNF182-mediated MFN2 ubiquitination. HK-2 and NRK-49F cells were co-transfected with Flag-MFN2, HA-Ub, and increasing amounts of Myc-tagged RNF182 (Myc-RNF182) as indicated. Cells were treated with MG132 and TGF-β1 ± rhIL-1Ra as shown. Flag-MFN2 was immunoprecipitated (IP: Flag), and ubiquitination was detected by Western blot analysis using anti-HA antibody. Input lysates were blotted for Myc-RNF182, Flag-MFN2, and GAPDH. Blots are representative of three independent experiments, and the values below the bands indicate the mean relative densitometric intensity (*n* = 3) normalized to GAPDH. Approximate molecular weight markers (kDa) are indicated.

Given that RNF182 is an E3 ubiquitin ligase whose expression is regulated by TGF-β1 and rhIL-1Ra, we sought to identify its relevant substrates in the context of kidney fibrosis. Bioinformatic analysis using protein interaction databases predicted MFN2 as a potential interacting partner and substrate of RNF182 (Fig. 5A). To experimentally validate this interaction, we performed co-immunoprecipitation (Co-IP) assays. Endogenous RNF182 was successfully immunoprecipitated with endogenous MFN2, confirming their physical association under basal conditions (Fig. 5B). We next investigated whether this interaction is regulated by TGF-β1 and rhIL-1Ra. Co-IP experiments revealed that TGF-β1 stimulation enhanced the interaction between RNF182 and MFN2 compared to control, whereas co-treatment with rhIL-1Ra attenuated the TGF-β1-induced increase in RNF182–MFN2 binding in both HK-2 and NRK-49F cells (Fig. 5C). To determine whether MFN2 is regulated at the transcriptional level, we assessed MFN2 mRNA expression by qRT-PCR. The results showed that neither TGF-β1, rhIL-1Ra, nor RNF182 overexpression significantly affected MFN2 mRNA levels in either cell line (Fig. S3A), suggesting that MFN2 is regulated primarily at the post-transcriptional level, likely through protein stability. Since E3 ligases typically target substrates for degradation, we evaluated whether RNF182 affects MFN2 protein stability. Using cycloheximide (CHX) chase assays, we monitored MFN2 protein levels after blocking new protein synthesis. In TGF-β1-treated cells, knockdown of RNF182 (shRNF182) slowed the degradation rate of MFN2 compared to control shRNA (shNC), indicating that endogenous RNF182 promotes MFN2 degradation under these conditions. Conversely, in cells treated with TGF-β1 plus rhIL-1Ra, overexpression of RNF182 accelerated MFN2 degradation compared to control vector (NC), suggesting that RNF182 levels are rate-limiting for MFN2 turnover (Fig. 5D). Consistent with these findings, Western blot analysis demonstrated that rhIL-1Ra treatment increased MFN2 protein levels in TGF-β1-stimulated cells, while subsequent RNF182 overexpression reversed this effect, reducing MFN2 protein back toward levels observed with TGF-β1 alone (Fig. S3B).To determine if RNF182-mediated MFN2 degradation occurs via the ubiquitin-proteasome system, we treated RNF182-overexpressing cells (under TGF-β1+rhIL-1Ra) with the proteasome inhibitor MG132. MG132 treatment prevented the RNF182-induced reduction in MFN2 protein, indicating that RNF182 promotes proteasomal degradation of MFN2 (Fig. 5E). We next directly assessed MFN2 ubiquitination using in vivo ubiquitination assays by co-transfecting Flag-tagged MFN2 and HA-tagged ubiquitin (HA-Ub). In the presence of MG132 to block proteasomal degradation, TGF-β1 stimulation increased the level of ubiquitinated Flag-MFN2 compared to control. Co-treatment with rhIL-1Ra markedly reduced this TGF-β1-induced MFN2 ubiquitination in both HK-2 and NRK-49F cells (Fig. 5F). To further confirm that RNF182 directly mediates MFN2 ubiquitination and that this process is modulated by TGF-β1/rhIL-1Ra, we co-transfected Myc-tagged RNF182, Flag-MFN2, and HA-Ub. Overexpression of Myc-RNF182 significantly enhanced the ubiquitination of Flag-MFN2. This RNF182-mediated ubiquitination was further promoted by TGF-β1 and was significantly reduced by rhIL-1Ra, even in the presence of overexpressed RNF182 (Fig. 5G). To assess the functional relevance of MFN2 regulation in this pathway, we tested whether restoring MFN2 expression could counteract the pro-fibrotic effects driven by RNF182 overexpression. In cells treated with TGF-β1+rhIL-1Ra + RNF182, co-overexpression of MFN2 significantly reduced the mRNA expression of pro-fibrotic markers (FN1, COL1A1, VIM, α-SMA) compared to cells overexpressing RNF182 alone in the same context (Fig. S3C). This suggests that the downregulation of MFN2 is a critical downstream event mediating the pro-fibrotic effects of RNF182. Taken together, these findings demonstrate that RNF182 interacts with MFN2, mediates its ubiquitination, and promotes its proteasomal degradation—a process enhanced by TGF-β1. rhIL-1Ra counteracts this by stabilizing MFN2 protein, likely via downregulation of RNF182. Moreover, the modulation of MFN2 levels appears to play a key functional role in regulating downstream fibrotic marker expression.

### The rhIL-1Ra-RNF182-MFN2 pathway regulates mitochondrial oxidative respiration

**Fig. 6:**
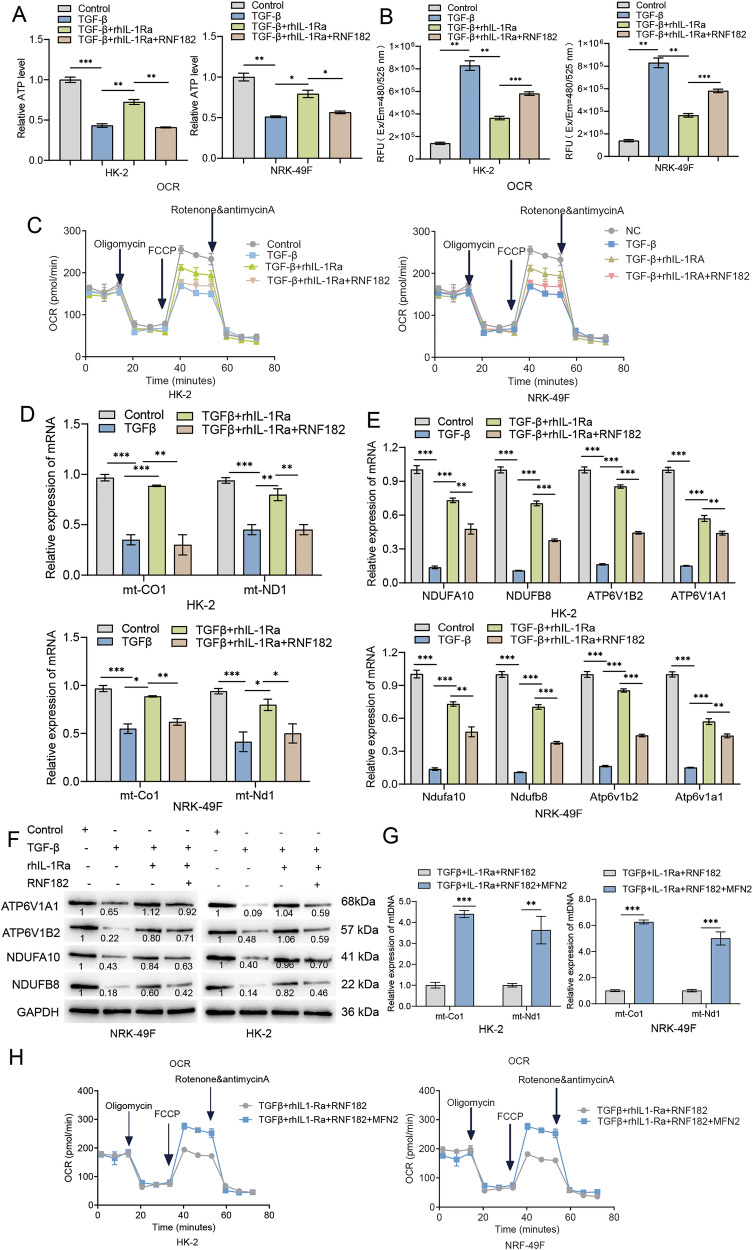
The rhIL-1Ra-RNF182 axis modulates mitochondrial function and gene expression in kidney cells. **A** Relative intracellular ATP levels in HK-2 (left) and NRK-49F (right) cells under the indicated conditions, measured using a luminescence-based ATP assay. Data normalized to the Control group. **B** ROS levels measured fluorescence in HK-2 (left) and NRK-49F (right) cells under the indicated conditions. Data presented as Relative Fluorescence Units (RFU, Ex/Em = 480/525 nm). **C** Real-time OCR profiles measured using the Seahorse XF Analyzer Mitochondrial Stress Test in HK-2 (left) and NRK-49F (right) cells under the indicated conditions. Arrows indicate sequential injections of oligomycin, FCCP, and rotenone/antimycin A. **D** Relative mRNA expression levels of mitochondrial DNA-encoded genes (*mt-Co1*, *mt-Nd1*) in HK-2 (top) and NRK-49F (bottom) cells under the indicated conditions, measured by qRT-PCR. Data normalized to GAPDH/Gapdh and shown relative to the Control group. **E** Relative mRNA expression levels of nuclear-encoded genes involved in cellular energy/ATP handling (Ndufa10, Ndufb8, Atp6v1b2, Atp6v1a1) in HK-2 (top) and NRK-49F (bottom) cells under the indicated conditions, measured by qRT-PCR. Data normalized to GAPDH/Gapdh and shown relative to the Control group. **F** Representative Western blot analysis showing protein levels of selected nuclear-encoded genes involved in cellular energy/ATP handling (ATP6V1A1, ATP6V1B2, NDUFA10, NDUFB8) in NRK-49F (left) and HK-2 (right) cell lysates under the indicated conditions. Blots are representative of three independent experiments, and the values below the bands indicate the mean relative densitometric intensity (*n* = 3) normalized to GAPDH. Approximate molecular weights (kDa) are indicated. **G** Rescue of mtDNA-encoded gene expression by MFN2. Relative mRNA levels of mt-Co1 and mt-Nd1 in HK-2 (left) and NRK-49F (right) cells co-overexpressing RNF182 and MFN2, demonstrating that MFN2 restoration reverses the suppressive effect of RNF182. **H** Functional rescue of mitochondrial respiration by MFN2. Real-time OCR profiles from a Seahorse Mitochondrial Stress Test in HK-2 (left) and NRK-49F (right) cells. The graph shows that co-overexpression of MFN2 restores the mitochondrial respiration deficits induced by RNF182 overexpression. Data are presented as mean ± SEM Statistical significance was determined using One-way ANOVA followed by Tukey’s multiple comparison post-hoc test. *p < 0.05, **p < 0.01, ***p < 0.001.

Given that MFN2, regulated by the rhIL-1Ra–RNF182 pathway, is a key protein involved in mitochondrial fusion and function, we investigated whether this axis influences mitochondrial bioenergetics in HK-2 and NRK-49F cells. Consistent with a role in mitochondrial regulation, initial experiments showed that TGF-β1 stimulation significantly reduced intracellular ATP levels (Fig. S4A) and increased ROS production (Fig. S4B) compared to control cells. Treatment with rhIL-1Ra partially but significantly reversed these effects, restoring ATP levels and reducing ROS (Fig. S4A, S4B). Furthermore, Seahorse XF analysis revealed that TGF-β1 decreased basal oxygen consumption rate (OCR), ATP-linked respiration, and maximal respiratory capacity in both cell lines, indicating impaired mitochondrial oxidative phosphorylation. Co-treatment with rhIL-1Ra partially rescued these parameters, elevating them toward control values (Fig. S4C). We next examined the role of RNF182 in this process. Overexpression of RNF182 in the presence of TGF-β1 and rhIL-1Ra significantly negated the protective effects of rhIL-1Ra, resulting in decreased ATP levels (Fig. 6A), increased ROS (Fig. 6B), and impaired mitochondrial respiration (both basal and maximal OCR) (Fig. 6C). Corresponding with these functional changes, TGF-β1 markedly downregulated the mRNA expression of mitochondrial DNA-encoded genes (mt-Co1, mt-Nd1) (Fig. 6D) nuclear‑encoded genes involved in cellular energy/ATP handling (Ndufa10, Ndufb8, Atp6v1a1, Atp6v1b2) (Fig. 6E) in both HK-2 and NRK-49F cells. rhIL-1Ra treatment significantly attenuated these decreases, while RNF182 overexpression abrogated the protective effects of rhIL-1Ra, further reducing the expression of these mitochondrial genes (Fig. 6D, E).To determine if mitochondrial gene expression was similarly affected in vivo, we analyzed kidney tissues from our animal models. Both UUO and 5/6Nx models exhibited substantial reductions in the expression of mtDNA-encoded genes (mt-Co1, mt-Nd1) (Fig. S4D) and nuclear‑encoded genes involved in cellular energy/ATP handling (Ndufa10, Ndufb8, Atp6v1a1, Atp6v1b2) (Fig. S4E). Treatment with rhIL-1Ra significantly restored the expression of these genes in both models (Fig. S4D, S4E), supporting the in vivo relevance of rhIL-1Ra’s effect on mitochondrial integrity. Protein analysis further supported these findings. Western blot analysis demonstrated that TGF-β1 reduced the protein levels of selected nuclear‑encoded genes involved in cellular energy/ATP handling (ATP6V1A1, ATP6V1B2, NDUFA10, NDUFB8), while rhIL-1Ra treatment partially restored these protein levels. Overexpression of RNF182 reversed this effect, diminishing the recovery conferred by rhIL-1Ra (Fig. 6F). To definitively establish that the RNF182-mediated loss of MFN2 is the direct cause of this mitochondrial collapse, we performed a critical rescue experiment. We tested whether re-introducing MFN2 could counteract the detrimental effects of RNF182 overexpression. Strikingly, restoring MFN2 levels was sufficient to rescue the mitochondrial deficits. Specifically, MFN2 co-overexpression substantially restored the expression of mtDNA-encoded genes (mt-Co1, mt-Nd1) that had been suppressed by RNF182 (Fig. 6G). Even more compellingly, at the functional level, MFN2 re-introduction fully rescued mitochondrial respiration, significantly improving both basal and maximal oxygen consumption rates (OCR) in both cell lines (Fig. 6H). Taken together, these rescue experiments provide the definitive causal evidence that the RNF182-MFN2 axis is a functionally critical regulator of mitochondrial health in our model. Restoration of MFN2 levels is sufficient to rescue mitochondrial gene expression and function downstream of RNF182.

### rhIL-1Ra demonstrates therapeutic potential comparable to established drugs in kidney fibrosis models

**Fig. 7:**
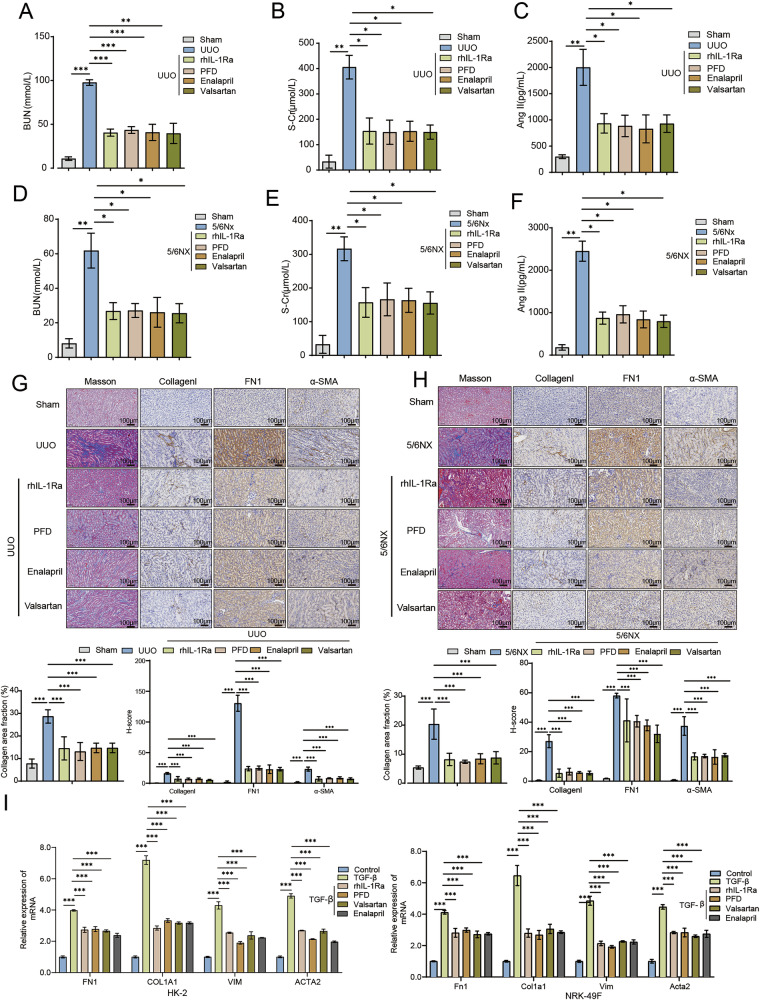
Benchmarking of rhIL-1Ra efficacy against established renal therapies in preclinical models of kidney injury and in vitro assays. Serum levels of BUN (**A**), S-Cr (**B**), and Ang-II (**C**) in mice from the UUO model groups at day 7 post-surgery. Serum levels of BUN (**D**), S-Cr (**E**), and Ang-II (**F**) in mice from the 5/6Nx model groups after treatment. **G** Representative histological (Masson’s Trichrome, First row) and immunohistochemical (Collagen I, FN1, α-SMA,) staining of kidney sections from Sham, UUO, and UUO mice treated with rhIL-1Ra, PFD, Enalapril, or Valsartan. Blue in Masson’s indicates collagen; brown in IHC indicates positive staining. Scale bars = 100 μm. **H** Representative histological (Masson’s Trichrome) and immunohistochemical (Collagen I, FN1, α-SMA) staining of kidney sections from Sham, 5/6Nx, and 5/6Nx mice treated with rhIL-1Ra, PFD, Enalapril, or Valsartan, with corresponding quantitative analysis shown below (bottom panels). Scale bars = 100 μm. **I** Relative mRNA expression levels of fibrotic marker genes (*COL1A1/Col1a1*, *FN1/Fn1*, *VIM/Vim*, *ACTA2/Acta2* [encoding α-SMA]) in HK-2 (left) and NRK-49F (right) cells treated with vehicle (Control), TGF-β1, or TGF-β1 + rhIL-1Ra, PFD, Valsartan, or Enalaprilat, measured by qRT-PCR. Data normalized to GAPDH/Gapdh and shown relative to the respective Control group. Data are presented as mean ± SEM. Statistical significance was determined using One-way ANOVA followed by Tukey’s multiple comparison post-hoc test. *p < 0.05, **p < 0.01, ***p < 0.001.

**Fig. 8:**
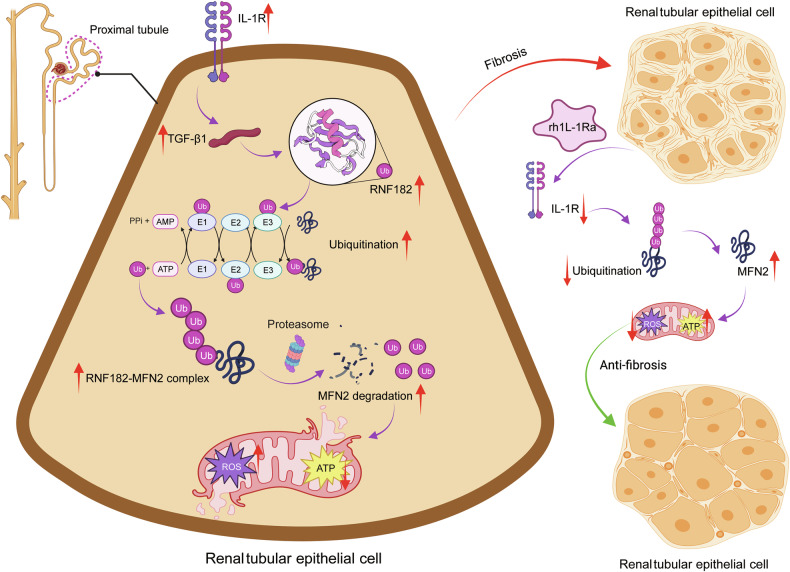
Schematic diagram of the proposed IL-1R/RNF182/MFN2 signaling pathway in renal fibrosis and the therapeutic mechanism of rhIL-1Ra. In the context of kidney injury, pro-inflammatory stimuli upregulate the expression of the Interleukin-1 Receptor (IL-1R) and its downstream effector, TGF-β1, in renal tubular epithelial cells. This leads to increased expression of the E3 ubiquitin ligase RNF182. RNF182, in turn, forms a complex with the mitochondrial fusion protein Mitofusin 2 (MFN2), targeting it for ubiquitination and subsequent proteasomal degradation. The depletion of MFN2 results in mitochondrial dysfunction, characterized by excessive reactive oxygen species (ROS) production and reduced ATP synthesis, which drives the progression of renal fibrosis. Therapeutic administration of recombinant human IL-1 receptor antagonist (rhIL-1Ra) blocks IL-1R signaling. This intervention suppresses the downstream upregulation of RNF182, thereby reducing MFN2 ubiquitination and degradation. The stabilization of MFN2 protein preserves mitochondrial function, mitigates oxidative stress, and restores cellular energy production, ultimately exerting a potent anti-fibrotic effect and protecting kidney tissue.

To evaluate the relative therapeutic potential of rhIL-1Ra against established anti-fibrotic and CKD treatments, we compared its effects with PFD, Enalapril, and Valsartan in both in vivo models and in vitro. In the UUO model, treatment with rhIL-1Ra significantly attenuated the increases in serum BUN (Fig. 7A), S-Cr (Fig. 7B), and Ang-II (Fig. 7C), similar to the effects observed with PFD, Enalapril, and Valsartan. All treatments showed significant improvement compared to the untreated UUO group, with rhIL-1Ra demonstrating efficacy within the range of these established agents in mitigating renal dysfunction and Ang-II elevation in this acute injury model. Similar comparative effects were observed in the 5/6Nx model of chronic kidney disease. rhIL-1Ra treatment significantly reduced the elevated serum BUN (Fig. 7D), S-Cr (Fig. 7E), and Ang-II (Fig. 7F) levels induced by 5/6Nx. The magnitude of these improvements was comparable to those achieved with PFD, Enalapril, and Valsartan treatment, indicating that rhIL-1Ra possesses significant reno-protective effects in this chronic model as well (Fig. 7D–F). Histological comparisons further supported these findings. In the UUO model, Masson’s trichrome staining and immunohistochemistry for Collagen I, FN1, and α-SMA revealed substantial fibrosis in untreated animals. Treatment with rhIL-1Ra markedly reduced collagen deposition and the expression of these fibrotic markers, achieving an attenuation comparable to that observed with PFD, Enalapril, and Valsartan (Fig. 7G). Likewise, in the 5/6Nx model, rhIL-1Ra treatment effectively reduced the extent of interstitial fibrosis and the expression of Collagen I, FN1, and α-SMA, with its efficacy appearing similar to the reference drugs (Fig. 7H). Finally, we compared the effects in vitro. TGF-β1 stimulation significantly upregulated the mRNA expression of Col1a1, Fn1, and α-Sma in both HK-2 and NRK-49F cells. Co-treatment with rhIL-1Ra significantly suppressed this upregulation. PFD, Enalapril, and Valsartan also inhibited the TGF-β1-induced expression of these fibrotic markers, and the inhibitory effect of rhIL-1Ra was comparable to these agents under the tested conditions (Fig. 7I). Collectively, these comparative analyses across different models (in vivo UUO and 5/6Nx, and in vitro) demonstrate that rhIL-1Ra exerts significant anti-fibrotic and reno-protective effects. The efficacy observed with rhIL-1Ra was comparable to that of established therapeutic agents like PFD, Enalapril, and Valsartan in mitigating kidney dysfunction, histological fibrosis, and the expression of pro-fibrotic markers in these experimental settings, suggesting its potential value as a therapeutic strategy for kidney fibrosis. A schematic model summarizing the proposed mechanism, wherein rhIL-1Ra exerts these reno-protective effects by inhibiting the IL-1R/RNF182/MFN2 signaling axis to prevent mitochondrial dysfunction and fibrosis, is presented in Fig. 8.

## Discussion

This study demonstrates the significant renoprotective potential of recombinant human interleukin-1 receptor antagonist (rhIL-1Ra) in mitigating both acute and chronic kidney injury, underpinned by a novel mechanistic pathway involving the E3 ubiquitin ligase RNF182 and the mitochondrial protein mitofusin 2 (MFN2). Our key findings reveal that rhIL-1Ra exerts robust anti-fibrotic effects in vivo across distinct murine models of kidney disease (UUO and 5/6Nx), preserving renal function. Mechanistically, we establish that rhIL-1Ra directly counteracts TGF-β1-induced pro-fibrotic activation in renal tubular epithelial cells and fibroblasts. Central to this effect is the identification of RNF182 as a critical downstream mediator, upregulated by TGF-β1 signaling but suppressed by rhIL-1Ra. We further show that RNF182 targets MFN2 for ubiquitination and proteasomal degradation. Consequently, rhIL-1Ra treatment stabilizes MFN2, thereby preserving mitochondrial function—a crucial component often compromised during fibrotic processes. Notably, comparative analyses indicate that rhIL-1Ra’s efficacy in these preclinical models is comparable to established therapies such as pirfenidone and RAS inhibitors, highlighting its therapeutic relevance.

Our findings significantly advance the understanding of IL-1R signaling in kidney fibrosis, moving beyond its established role predominantly discussed in the context of inflammation [30]. While previous studies using agents like Anakinra (a form of rhIL-1Ra) have suggested therapeutic benefits in certain kidney injury scenarios, these effects were often attributed broadly to systemic or local anti-inflammatory actions [31], sometimes lacking deep mechanistic insights into direct anti-fibrotic pathways within resident kidney cells. This study distinguishes itself by demonstrating potent anti-fibrotic efficacy not only in acute injury but critically, also in a progressive chronic kidney disease model (5/6Nx), and distinctly links IL-1R antagonism directly to the modulation of core fibrotic signaling cascades initiated by TGF-β1 within key renal cell types (epithelial cells and fibroblasts).

A key advancement of our work is the identification of RNF182 as a novel pro-fibrotic mediator in the kidney, positioned downstream of TGF-β1 signaling and negatively regulated by rhIL-1Ra. While RNF182 has been characterized in other biological contexts, its specific function in mediating renal fibrosis and its regulation by the interplay between IL-1R and TGF-β1 pathways represents a previously unrecognized connection [32]. Furthermore, prior research on Mitofusin 2 (MFN2) in kidney disease has largely focused on its crucial roles in mitochondrial dynamics, mitophagy, and overall mitochondrial health, often examining its transcriptional regulation or links to oxidative stress [33]. Our study provides novel mechanistic detail by uncovering a specific post-translational regulatory mechanism: RNF182-mediated ubiquitination and degradation of MFN2, dynamically controlled by pro-fibrotic stimuli (TGF-β1) and therapeutic intervention (rhIL-1Ra).

Crucially, unlike previous reports that might describe mitochondrial dysfunction as merely associated with fibrosis, our work explicitly connects the upstream IL-1R signaling pathway, via the RNF182-MFN2 axis, directly to the preservation of mitochondrial bioenergetics (ATP production, respiratory capacity, ROS levels). We demonstrate that protecting MFN2 stability is functionally required for mitigating mitochondrial damage and downstream fibrotic responses within this signaling framework. This provides a more precise and actionable mechanistic insight compared to generalized descriptions of IL-1 blockade improving mitochondrial health [34]. Finally, while established therapies like RAS inhibitors and Pirfenidone target different pathways [35], our benchmarking results position rhIL-1Ra as comparably effective in preclinical models, but highlighting its distinct mechanism of action focused on IL-1R/RNF182/MFN2/mitochondria, suggesting a potentially complementary or alternative therapeutic strategy, particularly given Anakinra’s established clinical safety profile in other indications.

Despite the promising findings, this study has several limitations. Firstly, our use of systemic administration of rhIL-1Ra in vivo cannot definitively distinguish between the drug’s direct effects on resident kidney cells and its indirect benefits from modulating systemic inflammation. While our comprehensive in vitro data strongly support a direct, kidney-specific mechanism, some of the observed renoprotection may also derive from global anti-inflammatory effects, such as reduced infiltration of circulating immune cells. Future studies employing kidney-targeted drug delivery or cell type–specific conditional knockouts of IL-1R would be invaluable to dissect these distinct contributions. Secondly, while the UUO and 5/6Nx models are widely used and recapitulate key features of human kidney fibrosis and CKD progression respectively, they do not fully encompass the heterogeneity and complexity of human kidney disease etiologies (e.g., diabetic nephropathy, hypertensive nephrosclerosis). The efficacy of rhIL-1Ra should be validated in a broader range of preclinical models. Thirdly, our mechanistic investigation primarily focused on tubular epithelial cells and fibroblasts; the effects of rhIL-1Ra on other crucial renal cell types, such as podocytes, endothelial cells, and resident or infiltrating immune cells, warrant further investigation. Fourth, while we identified the RNF182-MFN2 axis, the precise signaling events connecting IL-1R activation to RNF182 transcriptional regulation remain to be elucidated. Furthermore, whether RNF182 targets other relevant substrates besides MFN2 in this context is unknown. Finally, the current study assessed relatively short-term outcomes; long-term efficacy, potential off-target effects, optimal dosing regimens, and the pharmacokinetics/pharmacodynamics of rhIL-1Ra specifically in the context of CKD require thorough investigation in future studies. Lastly, the preclinical benchmarking provides valuable context but cannot directly predict relative efficacy in human patients, which requires rigorous clinical trials.

To address these limitations and build upon our findings, several approaches can be proposed. Indeed, as a crucial first step towards establishing clinical relevance, we performed a series of validation experiments using primary Human Renal Proximal Tubular Epithelial Cells (HRPTEpiC). Reassuringly, these experiments confirmed that the core signaling axis identified in our study is conserved and functional in human primary cells (all data in Fig. S5). Specifically, we demonstrated that rhIL-1Ra effectively counteracted the TGF-β1-induced upregulation of key fibrotic markers in these cells. Critically, this anti-fibrotic effect was associated with the same mechanistic signature observed in our main studies: the induction of the E3 ligase RNF182 by TGF-β1 was suppressed by rhIL-1Ra.While this new data provides a vital bridge to human pathophysiology and substantially strengthens the clinical relevance of our work, future investigations should validate the role of the RNF182-MFN2 axis in more diverse preclinical models of CKD, potentially including models of diabetic nephropathy or employing human kidney organoids or precision-cut kidney slices for enhanced clinical relevance. Key findings should be further confirmed using additional primary human kidney cells, including tubular epithelial cells, fibroblasts, and potentially podocytes and endothelial cells, possibly utilizing co-culture systems to better mimic cellular interactions. Employing unbiased proteomic or ubiquitinomic approaches could provide a broader view of RNF182 substrates and other pathways modulated by rhIL-1Ra treatment in the context of kidney injury. Investigating the potential interplay between the IL-1R/RNF182 pathway and canonical TGF-β1/Smad signaling would also be informative. Furthermore, exploring optimized delivery strategies or formulations for rhIL-1Ra specifically for kidney disease could enhance its therapeutic index.

Based on our results, we propose several new hypotheses and future research directions. We hypothesize that direct inhibition of RNF182 itself, potentially through small molecule inhibitors or targeted genetic approaches in vivo, could represent a novel therapeutic strategy for kidney fibrosis, possibly bypassing the need for broader IL-1R blockade. Understanding whether specific patient subgroups (e.g., those with high IL-1 pathway activation markers or specific mitochondrial genetic backgrounds) might benefit more from rhIL-1Ra therapy could pave the way for personalized medicine approaches. Well-designed preclinical studies exploring combination therapies (e.g., rhIL-1Ra plus an ACE inhibitor or Pirfenidone) are highly warranted to assess potential synergistic benefits based on their distinct mechanisms. Ultimately, prospective clinical trials are necessary to evaluate the safety and efficacy of rhIL-1Ra (such as Anakinra) for treating human kidney fibrosis and CKD, potentially stratifying patients based on inflammatory or mitochondrial biomarkers.

In conclusion, this study provides compelling evidence for the reno-protective effects of rhIL-1Ra in experimental kidney disease. We identified a novel underlying mechanism involving the suppression of the E3 ligase RNF182, stabilization of MFN2, and preservation of mitochondrial function, linking IL-1 signaling directly to cellular bioenergetics and fibrotic control. With demonstrated preclinical efficacy comparable to existing treatments and a distinct mechanism of action, rhIL-1Ra represents a promising therapeutic candidate for kidney fibrosis, meriting further investigation towards clinical application, potentially offering a new strategy to combat the progression of chronic kidney disease.

## Materials and methods

### Unilateral ureteral obstruction (UUO) model

Mice have been anesthetized with isoflurane (Baxter, Cat# 10019-352-60). Under sterile conditions, the left ureter was exposed via a flank incision and completely ligated using two 4-0 silk sutures (Ethicon, Cat# K870H). Sham-operated mice underwent the same surgical procedure but without ureteral ligation.

### 5/6 nephrectomy (5/6Nx) model

A two-step procedure was performed. First, under isoflurane anesthesia, approximately two-thirds of the left kidney was surgically removed after ligation of the renal artery branches supplying that portion. One week later, a right total nephrectomy was performed via a separate flank incision. Sham-operated mice underwent sham surgeries at both time points without removal of kidney tissue.

On the distinctive experimental endpoints, mice were euthanized for tissue and blood collection.

A portion of the kidney tissue was fixed in 10% neutral buffered formalin for histological evaluation, whilst the remaining tissue was snap-frozen in liquid nitrogen and stored at –80 °C for subsequent molecular and biochemical assays.

### Reagents and drug treatments

Recombinant human IL-1Ra (rhIL-1Ra) turned into purchased from (R&D Systems, Cat# 280-RA). Recombinant human TGF-β1 became received from (R&D Systems, Cat# 240-B-002/CF). Pirfenidone (PFD) (Selleckchem, Cat# S2907), Enalapril maleate (Sigma-Aldrich, Cat# E6888), Valsartan (Selleckchem, Cat# S1894), MG132 (Selleckchem, Cat# S2619), and Cycloheximide (CHX) (Sigma-Aldrich, Cat# C7698) had been bought from the indicated suppliers.

#### In vivo treatments

To assess the therapeutic potential of the compounds, all treatments were initiated after the induction of kidney injury. For the UUO model, administration started immediately post-surgery on day 0 and continued daily until sacrifice on day 7 or 14. For the 5/6Nx model, a model of established chronic injury, treatments began one week after the final nephrectomy and continued for 4 weeks.

Mice were randomly assigned to the following treatment groups:

#### Control

Received daily oral gavage of the vehicle solution sterile water and intraperitoneal (i.p.) injections of sterile saline on the same schedule as the corresponding drug treatments.

#### rhIL-1Ra

Administered via i.p. injection at a dose of 100 mg/kg/day1

#### Pirfenidone (PFD)

Administered via oral gavage at 300 mg/kg/day, dissolved in sterile water.

#### Enalapril

Administered in the drinking water at a concentration of 100 mg/L.

#### Valsartan

Administered via oral gavage at 10 mg/kg/day, dissolved in sterile water.

#### In Vitro remedies

Cells were serum-starved for 12–24 h earlier than stimulation. TGF-β1 was used at a concentration of (10 ng/mL,24 h). rhIL-1Ra became introduced 1 h prior to TGF-β1 stimulation at a concentration of 100 ng/mL. PFD (500 μM), Enalaprilat (10 μM), and Valsartan (10 μM) had been introduced concurrently with or 1 h previous to TGF-β1 as indicated. MG132 (10 μM) or CHX (200 μg/mL) were delivered as described for specific assays.

### Cell culture

Human kidney proximal tubular epithelial cells (HK-2, ATCC CRL-2190) were cultured in DMEM/F-12 medium (Gibco, Cat# 11330032) supplemented with 10% fetal bovine serum (FBS) (Gibco, Cat# 10270106) and 1% penicillin-streptomycin (Gibco, Cat# 15140122). Rat kidney fibroblasts (NRK-49F, ATCC CRL-1570) had been cultured in DMEM high glucose (Gibco, Cat# 11965092) supplemented with 5% FBS and 1% penicillin-streptomycin. Both cell lines had been maintained at 37 °C in a humidified atmosphere containing 5% CO₂. Primary Human Renal Proximal Tubular Epithelial Cells (HRPTEpiC) were purchased from ScienCell Research Laboratories (Cat#4100-SC, Carlsbad, CA, USA). Cells were cultured in Epithelial Cell Medium (EpiCM, 4101, ScienCell) supplemented with 2% fetal bovine serum, 1% Epithelial Cell Growth Supplement (EpiCGS), and 1% penicillin/streptomycin solution, according to the manufacturer’s instructions. HRPTEpiC were maintained at 37 °C in a humidified atmosphere containing 5% CO₂ and were used for experiments between passages 2 and 5.

All cell lines were recently authenticated by Short Tandem Repeat (STR) profiling and routinely tested negative for mycoplasma contamination using a PCR-based assay.

### Histological analysis and immunohistochemistry (IHC)

Kidney tissues had been steady in 4% paraformaldehyde (PFA), dehydrated via graded ethanol series, cleared in xylene, and embedded in paraffin. Sections (4 μm) had been stained with Masson’s Trichrome the usage of a standard kit (Sigma-Aldrich, Cat# HT15) to assess collagen deposition. For IHC, sections have been deparaffinized, rehydrated, and subjected to antigen retrieval the use of citrate buffer (pH 6.0) (Vector Labs, Cat# H-3300) in a microwave oven.

Endogenous peroxidase activity became quenched with 3% H₂O₂.Sections were blocked with 5% normal goat serum (Vector Labs, Cat# S-1000) and incubated in a unmarried day at 4 °C with primary antibodies against: Collagen I (Abcam, Cat# ab34710, 1 : 200 dilution), Fibronectin 1 (FN1) (Abcam, Cat# ab2413, 1 : 200 dilution), α-Smooth Muscle Actin (α-SMA) (Sigma-Aldrich, Cat# A2547, 1 : 400 dilution), or RNF182 (Abcam, Cat# 72533, 1 : 100 dilution).After washing, sections have been incubated with a biotinylated secondary antibody (Vector Labs Goat Anti-Rabbit IgG (H + L), Cat# BA-1000) observed by means of using avidin-biotin-peroxidase complex (ABC kit) (Vector Labs, Cat# PK-6100), and visualized the usage of 3,3’-diaminobenzidine (DAB) (Vector Labs, Cat# SK-4105). Sections were counterstained with hematoxylin. Images had been captured using a bright-field microscope (Olympus BX53) equipped with a digital camera. Quantification of stained areas was completed using ImageJ software (NIH) on at least 10 randomly selected fields per kidney section (original magnification, × 400).

### RNA isolation and quantitative real-time PCR (qRT-PCR)

Total RNA was extracted from kidney tissues or cultured cells using the RNeasy Mini Kit (Qiagen, Cat# 74104) or TRIzol Reagent (Invitrogen, Cat# 15596026CN) according to the manufacturer’s instructions. cDNA was synthesized from 1 μg of total RNA using the iScript cDNA Synthesis Kit (Bio-Rad, Cat# 1708891). qRT-PCR was performed using SsoAdvanced Universal SYBR Green Supermix (Bio-Rad, Cat# 1725271) on a CFX96 Real-Time PCR Detection System (Bio-Rad). Relative mRNA expression was calculated using the 2^-ΔΔCt method, normalized to the housekeeping gene Gapdh (for mouse and rat) or GAPDH (for human). The sequences of all primers used in this study are listed in Supplementary Table 1.

### Protein extraction and western blot analysis

Kidney tissues or cells were lysed in ice-cold RIPA buffer (Cell Signaling Technology (CST), Cat# 9806) supplemented with protease inhibitor cocktail (Roche, Cat# 11836153001) and phosphatase inhibitor cocktail (Roche, Cat# 4906845001). Protein concentrations were determined using the Pierce BCA Protein Assay Kit (Thermo Fisher Scientific, Cat# 23225). Equal amounts of protein (20–40 μg) were separated by SDS-PAGE (8–12% gels) and transferred onto polyvinylidene difluoride (PVDF) membranes (Millipore, Cat# IPVH00010). Membranes were blocked with 5% non-fat dry milk or 5% bovine serum albumin (BSA) in Tris-buffered saline containing 0.1% Tween-20 (TBST) for 1 h at room temperature. Membranes were then incubated overnight at 4 °C with primary antibodies diluted in blocking buffer. GAPDH as the loading control. All primary antibodies used for this study are detailed in Supplementary Table 2. After washing with TBST, membranes were incubated with horseradish peroxidase (HRP)-conjugated secondary antibodies (CST Goat anti-Rabbit IgG HRP, Cat# 7074 or Goat anti-Mouse IgG HRP, Cat# 7076, 1:2000-1:5000) for 1 h at room temperature. Protein bands were visualized using enhanced chemiluminescence (ECL) reagents (Thermo Fisher Scientific, SuperSignal West Pico PLUS, Cat# 34580) and imaged using a chemiluminescence detection system (Bio-Rad ChemiDoc MP). Band intensities were quantified using ImageJ or manufacturer’s software.

### Immunofluorescence staining (IF)

Cells grown on coverslips were fixed with 4% PFA for 15 min, permeabilized with 0.2% Triton X-100 in PBS for 10 min, and blocked with 5% BSA in PBS for 1 h. Cells were incubated with primary antibodies against IL-1R (R&D Systems, Cat# AF269, 1:100 dilution) overnight at 4 °C. After washing, cells were incubated with Alexa Fluor-conjugated secondary antibodies (Invitrogen Goat anti-Mouse IgG Alexa Fluor 594, Cat# A11032, 1:500 dilution) for 1 h at room temperature. Nuclei were counterstained with DAPI (Invitrogen, Cat# D1306). Coverslips were mounted using ProLong Gold Antifade Mountant (Invitrogen, Cat# P36930). Images were acquired using a confocal laser scanning microscope (Zeiss LSM 880 or Leica SP8).

### RNA sequencing (RNA-seq) and data analysis

Total RNA turned into extracted from HK-2 cells treated with Control, TGF-β1, or TGF-β1 + rhIL-1Ra (*n* = 3).RNA quality became assessed the use of an Agilent 2100 Bioanalyzer (RIN > 8.0). Library preparation become achieved using the NEBNext Ultra II RNA Library Prep Kit for Illumina (NEB, Cat# E7770) following the manufacturer’s instructions. Sequencing was performed on an Illumina NovaSeq 6000 platform producing 150 bp paired-end reads to a depth of approximately 30–50 million reads consistent with sample. Raw reads have been processed using FastQC for quality control. Reads have been aligned to the human reference genome (GRCh38) using STAR aligner (v2.7.9a). Gene counts had been quantified the use of featureCounts (v2.0.3). Differential gene expression evaluation was achieved the usage of DESeq2 (v1.36.0) package in R (v4.2.1) .Genes with an absolute Log2FoldChange ≥ 3 and an adjusted p-value (FDR < 0.05) have been considered differentially expressed. Venn diagrams were generated the use of (online Venny 2.1). Hierarchical clustering and heatmap visualization had been executed the use of (pheatmap package in R).

### Plasmid construction and transfection

Full-length human RNF182 and MFN2 cDNA were amplified by PCR from a human kidney cDNA library and cloned into expression vectors (pcDNA3.1(+) or variants with Flag/Myc tags) using standard restriction enzyme digestion and ligation or Gibson assembly. All constructs were verified by Sanger sequencing. Plasmids (including empty vector control) were transfected into HK-2 or NRK-49F cells using Lipofectamine 3000 Reagent (Invitrogen, Cat# L3000015) according to the manufacturer’s protocol. Overexpression efficiency was confirmed by qRT-PCR and Western blot. Plasmid encoding HA-tagged Ubiquitin (HA-Ub) was obtained from (Addgene Plasmid #18712).

### Small hairpin RNA (shRNA) interference

Lentiviral particles containing shRNA targeting rat Rnf182 (shRNF182) or a non-targeting control sequence (shCtrl) within the pLKO. 1 vector backbone have been purchased from (Sigma-Aldrich MISSION shRNA). NRK-49F cells have been transduced with lentiviral particles in the presence of polybrene (8 μg/mL, Sigma, Cat# H9268) and selected with puromycin (1–2 μg/mL, Gibco, Cat# A1113803) for 3–5 days. Knockdown efficiency became confirmed by means of qRT-PCR and Western blot.

### Cell migration assay

Cell migration was assessed using Transwell inserts (8.0 μm pore size) (Corning, Cat# 3422) in 24-well plates. Cells (HK-2 or NRK-49F, (Specify density, 5 × 10^4 cells/well)) suspended in serum-free medium were seeded into the upper chamber. The lower chamber contained medium with 10% FBS (as chemoattractant) or specific treatments (TGF-β1 ± rhIL-1Ra). After incubation for 24 h, non-migrated cells on the upper surface of the membrane were removed with a cotton swab. Migrated cells on the lower surface were fixed with 4% PFA and stained with 0.1% crystal violet. Images of migrated cells were captured from five random fields per insert. Migrated cells were counted, the crystal violet stain was eluted with 10% acetic acid.

### Co-immunoprecipitation (Co-IP) assay

Cells were lysed in ice-cold Co-IP lysis buffer (50 mM Tris-HCl pH 7.4, 150 mM NaCl, 1 mM EDTA, 1% NP-40 or Triton X-100, supplemented with protease inhibitors). Cell lysates (0.5-1 mg total protein) were pre-cleared with Protein A/G magnetic beads (Thermo Fisher Scientific, Cat# 88802) for 1 h at 4 °C. Pre-cleared lysates were incubated overnight at 4 °C with primary antibodies against endogenous RNF182, MFN2, or control IgG (Normal Rabbit IgG, CST, Cat# 2729). For tagged proteins, anti-Flag (Sigma, F1804) or anti-Myc (CST, 2276) antibodies were used. Antibody-protein complexes were captured by incubating with Protein A/G magnetic beads for 2–4 h at 4 °C. Beads were washed extensively (3–5 times) with ice-cold Co-IP lysis buffer. Immunoprecipitated proteins were eluted by boiling in SDS sample buffer and analyzed by Western blot using antibodies against the potential interacting partners.

### Ubiquitination assays

Cells have been lysed in ice-cold Co-IP lysis buffer (50 mM Tris-HCl pH 7.4, 150 mM NaCl, 1 mM EDTA, 1% NP-40 or Triton X-100, supplemented with protease inhibitors). Cell lysates (0.5–1 mg total protein) were pre-cleared with Protein A/G magnetic beads (Thermo Fisher Scientific, Cat# 88802) for 1 h at 4 °C. Pre-cleared lysates were incubated overnight at 4 °C with primary antibodies against endogenous RNF182, MFN2, or control IgG (Normal Rabbit IgG, CST, Cat# 2729). For tagged proteins, anti-Flag (Sigma, F1804) or anti-Myc (CST, 2276) antibodies had been used. Antibody-protein complexes were captured by incubating with Protein A/G magnetic beads for 2–4 h at 4 °C. Beads were washed extensively (3-5 times) with ice-cold Co-IP lysis buffer, including washes with high salt buffer if necessary. Immunoprecipitated proteins were eluted by boiling in SDS sample buffer and analyzed by Western blot using anti-HA antibody (to detect ubiquitinated MFN2) and anti-Flag antibody (to confirm MFN2 pulldown). Input lysates were also analyzed.

### Cycloheximide (CHX) chase assay

Cells have been treated as indicated (TGF-β1 ± shRNF182 or TGF-β1 + rhIL-1Ra ± RNF182 overexpression). CHX (200 μg/mL) was added to inhibit new protein synthesis. Cells have been harvested at different time points (0, 4, 8, 12 h) after CHX addition. Cell lysates were prepared, and MFN2 protein levels were analyzed by using Western blot, normalized to the loading control GAPDH. Protein half-life was estimated from the degradation curves.

### Measurement of intracellular ATP levels

Intracellular ATP levels had been measured the usage of the CellTiter-Glo® Luminescent Cell Viability Assay Kit (Promega, Cat# G7570) in line with the manufacturer’s instructions. Briefly, cells cultured in opaque-walled 96-well plates were lysed by adding the CellTiter-Glo® reagent. Luminescence, proportional to ATP concentration, was measured using a luminometer (Tecan Spark). Results had been normalized to total protein concentration or cell number determined in parallel wells.

### Cellular reactive oxygen species (ROS) assay (DCFH‑DA)

Cellular ROS were measured with a DCFH‑DA–based Reactive Oxygen Species Assay Kit (Beyotime; Cat. S0033S). HK‑2 and NRK‑49 F cells (50–70% confluence) received the indicated treatments (Control, TGF‑β1, ± rhIL‑1Ra, ± RNF182 OE). Cultures were incubated in situ with DCFH‑DA (10 µM; 1:1000 dilution in serum‑free, phenol‑red–free medium) for 30 min at 37 °C in the dark, rinsed twice with HBSS, and read immediately (Ex 480 nm / Em 525 nm) on a fluorescence plate reader. Dye‑free background was subtracted.

### Seahorse XF metabolic flux analysis

Mitochondrial respiration was assessed using a Seahorse XFe96 Analyzer (Agilent technology). HK-2 or NRK-49F cells were seeded into Seahorse XF96 cellular tradition microplates (1–2 × 10^4 cells/nicely) and dealt with as indicated. at the day of the assay, the lifestyle medium emerge as modified with Seahorse XF DMEM medium (pH 7.4) (Agilent, Cat# 103575-one hundred) supplemented with glucose (10 mM), pyruvate (1 mM), and glutamine (2 mM). The Mitochondrial pressure test emerge as finished with the resource of sequential injection of oligomycin (1.0–1.5 μM), FCCP (carbonyl cyanide-4-(trifluoromethoxy) phenylhydrazone) (1.0–1.5 μM), and a mixture of rotenone and antimycin A (0.5 μM each) (All from Agilent Seahorse XF cellular Mito pressure check kit, Cat# 103015-100). Oxygen consumption price (OCR) changed into measured in real-time. Basal respiratory, ATP-associated respiration, maximal breathing, and non-mitochondrial oxygen intake had been calculated in step with the manufacturer’s suggestions and Seahorse Wave software. records were normalized to popular protein content material cloth in keeping with well measured after the assay.

### Enzyme-linked immunosorbent assay (ELISA)

Concentrations of IL-1β, TNF-α, IL-10 in mouse serum or cell culture supernatants had been measured using commercially available ELISA kits (R&D Systems DuoSet ELISA kits, Cat# DY401 for IL-1β, DY410 for TNF-α, DY417 for IL-10) following the manufacturer’s protocols. Serum Angiotensin II (Ang II) levels had been measured using an Angiotensin II ELISA kit (Abcam, Cat# ab285306). Absorbance was read at 450 nm using a microplate reader.

### Statistical analysis

All experiments were conducted in a blinded manner where possible.

#### Image quantification

Densitometry of Western blot bands was quantified using ImageJ software (v1.54p; NIH). The intensity of each target protein was normalized to its corresponding GAPDH loading control and expressed as a fold change relative to the control group. For histological analysis, collagen area fraction (%) from Masson’s trichrome staining was also quantified using ImageJ (v1.54p; NIH) by calculating the percentage of blue-stained area. Immunohistochemistry (IHC) staining was quantified using QuPath [36] software (v0.6.0) to generate an H-score (0–300) based on the percentage of cells at different staining intensities. The results for collagen area fraction and H-score are presented as absolute values.

Data are presented as mean ± standard error of the mean (SEM) as indicated in the figure legends. Statistical analyses were performed using GraphPad Prism software (v.8.3.0, GraphPad Software, La Jolla, CA). Comparisons between two groups were performed using an unpaired two-tailed Student’s *t* test. Comparisons among three or more groups were performed using one-way analysis of variance (ANOVA) followed by Tukey’s or Bonferroni’s multiple comparison post-hoc test. Two-way ANOVA was used for experiments with two independent variables (treatment and time), followed by appropriate post hoc tests. A *p* value < 0.05 was considered statistically significant. The number of replicates (3) for each experiment.

## Supplementary information

Supplementary files

## Data Availability

The datasets used and analyzed during the current study are available from the corresponding author on reasonable request. The original Western blot data are provided in Supplementary Fig. S3.
