# Supplementary material for: IL‑1 receptor antagonism attenuates renal fibrosis via RNF182‑driven MFN2 destabilization and mitochondrial dysfunction
Source: Cell Death Discov. 2025 Dec 29;12:67. doi: 10.1038/s41420-025-02929-4 (PMC12848067; doi:10.1038/s41420-025-02929-4)
Supplement: Supplementary file 1 — Supplementary files [file 41420_2025_2929_MOESM1_ESM.pdf]

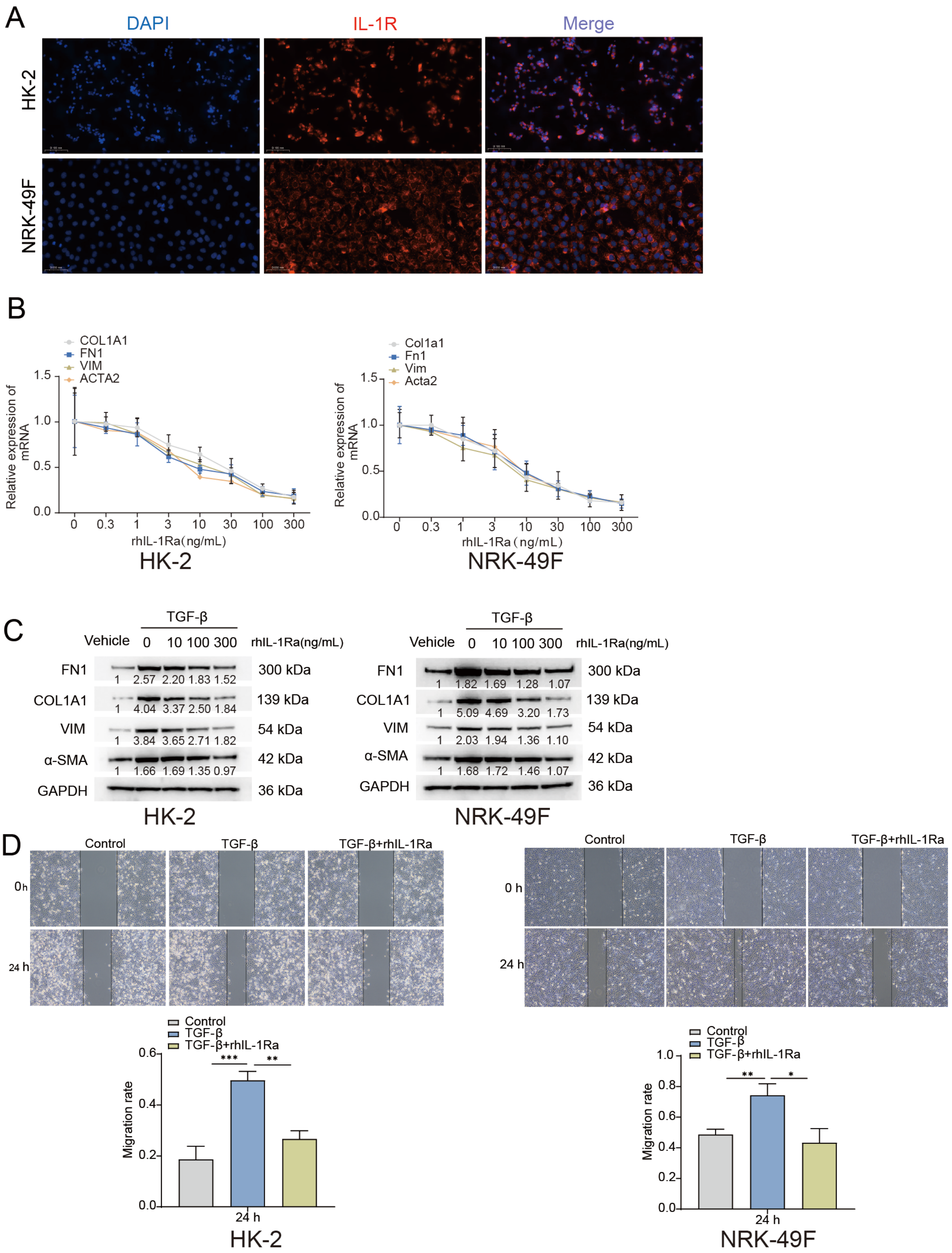
A
DAPI
IL-1R
Merge
HK-2
NRK-49F
B
COL1A1
FN1
VIM
ACTA2
Relative expression of mRNA
rhIL-1Ra(ng/mL)
HK-2
Col1a1
Fn1
Vim
Acta2
NRK-49F
C
TGF-β
Vehicle 0 10 100 300 rhIL-1Ra(ng/mL)
FN1 300 kDa
1 2.57 2.20 1.83 1.52
COL1A1 139 kDa
1 4.04 3.37 2.50 1.84
VIM 54 kDa
1 3.84 3.65 2.71 1.82
α-SMA 42 kDa
1 1.66 1.69 1.35 0.97
GAPDH 36 kDa
HK-2
FN1 300 kDa
1 1.82 1.69 1.28 1.07
COL1A1 139 kDa
1 5.09 4.69 3.20 1.73
VIM 54 kDa
1 2.03 1.94 1.36 1.10
α-SMA 42 kDa
1 1.68 1.72 1.46 1.07
GAPDH 36 kDa
NRK-49F
D
Control
TGF-β
TGF-β+rhIL-1Ra
0 h
24 h
Migration rate
24 h
HK-2
NRK-49F

Figure S1

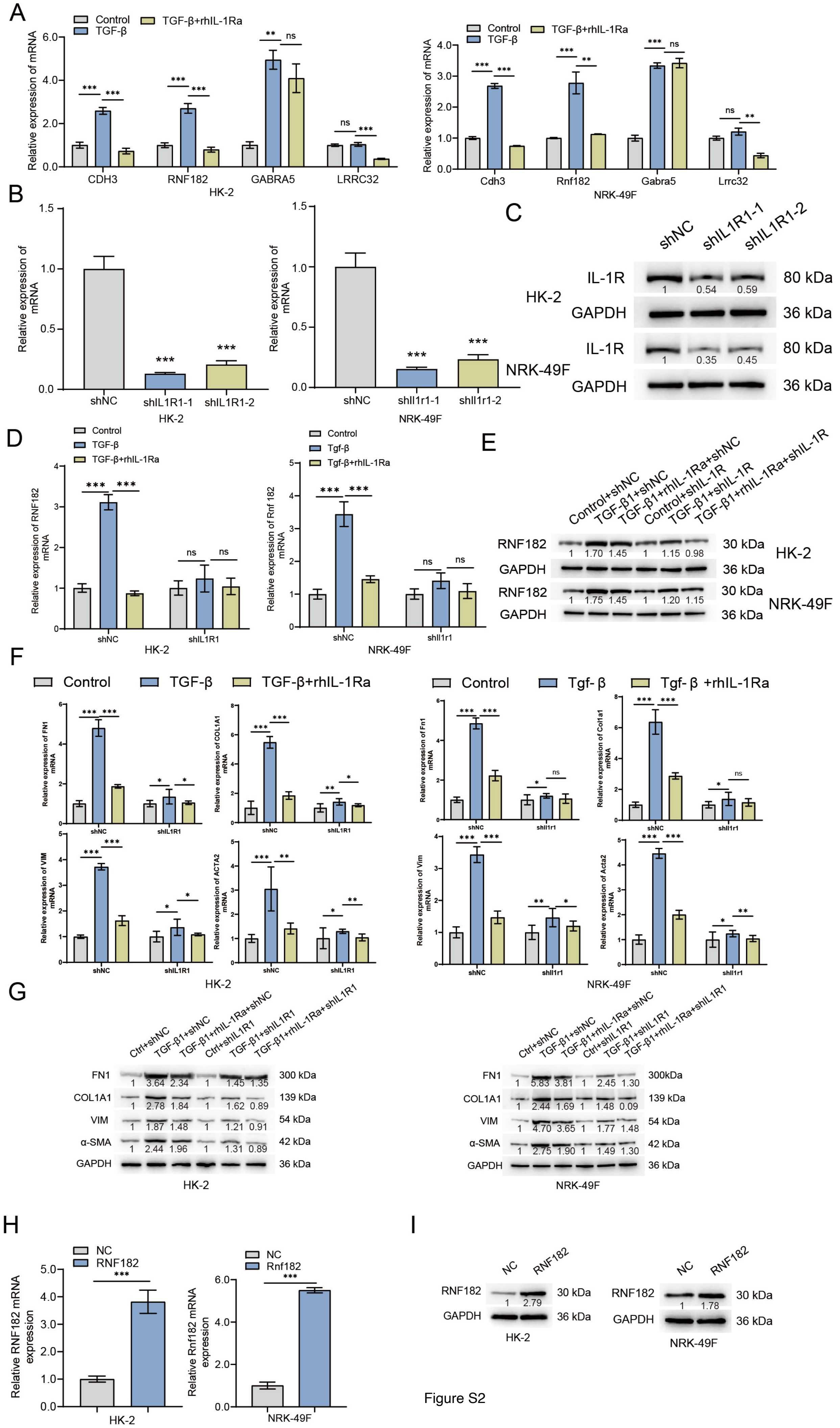
A
Control
TGF-β
TGF-β+rhIL-1Ra
Relative expression of mRNA
CDH3
RNF182
GABRA5
LRRC32
HK-2
Cdh3
Rnf182
Gabra5
Lrrc32
NRK-49F
B
Relative expression of mRNA
shNC
shIL1R1-1
shIL1R1-2
HK-2
shIl1r1-1
shIl1r1-2
NRK-49F
C
shNC
shIL1R1-1
shIL1R1-2
IL-1R
GAPDH
80 kDa
36 kDa
HK-2
NRK-49F
1
0.54
0.59
1
0.35
0.45
D
Control
TGF-β
TGF-β+rhIL-1Ra
Relative expression of RNF182 mRNA
shNC
shIL1R1
HK-2
Tgf-β
Tgf-β+rhIL-1Ra
Relative expression of Rnf 182 mRNA
shIl1r1
NRK-49F
E
Control+shNC
TGF-β1+shNC
TGF-β1+rhIL-1Ra+shNC
Control+shIL-1R
TGF-β1+shIL-1R
TGF-β1+rhIL-1Ra+shIL-1R
RNF182
GAPDH
30 kDa
36 kDa
1 1.70 1.45 1 1.15 0.98
1 1.75 1.45 1 1.20 1.15
HK-2
NRK-49F
F
Control
TGF-β
TGF-β+rhIL-1Ra
Tgf- β
Tgf- β +rhIL-1Ra
Relative expression of FN1 mRNA
Relative expression of COL1A1 mRNA
Relative expression of VIM mRNA
Relative expression of ACTA2 mRNA
Relative expression of Fn1 mRNA
Relative expression of Col1a1 mRNA
Relative expression of Vim mRNA
Relative expression of Acta2 mRNA
HK-2
NRK-49F
G
Ctrl+shNC
TGF-β1+shNC
TGF-β1+rhIL-1Ra+shNC
Ctrl+shIL1R1
TGF-β1+shIL1R1
TGF-β1+rhIL-1Ra+shIL1R1
FN1
COL1A1
VIM
α-SMA
GAPDH
300 kDa
139 kDa
54 kDa
42 kDa
36 kDa
1 3.64 2.34 1 1.45 1.35
1 2.78 1.84 1 1.62 0.89
1 1.87 1.48 1 1.21 0.91
1 2.44 1.96 1 1.31 0.89
HK-2
1 5.83 3.81 1 2.45 1.30
1 2.44 1.69 1 1.48 0.09
1 4.70 3.65 1 1.77 1.48
1 2.75 1.90 1 1.49 1.30
NRK-49F
H
NC
RNF182
Relative RNF182 mRNA expression
HK-2
Rnf182
Relative Rnf182 mRNA expression
NRK-49F
I
NC
RNF182
GAPDH
30 kDa
36 kDa
1 2.79
HK-2
1 1.78
NRK-49F

Figure S2

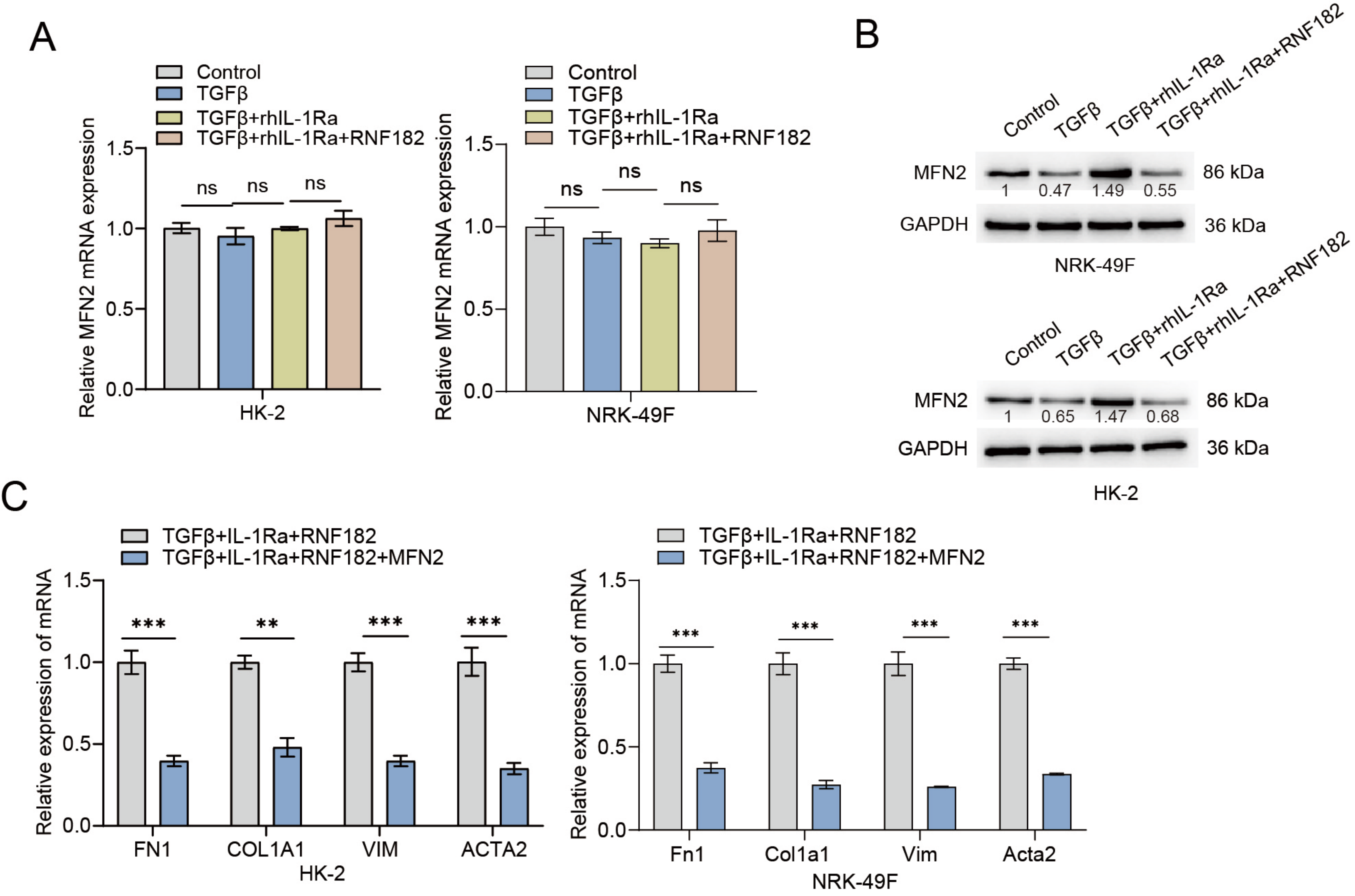

A
Control
TGFβ
TGFβ+rhIL-1Ra
TGFβ+rhIL-1Ra+RNF182
Relative MFN2 mRNA expression
ns
HK-2
NRK-49F
B
MFN2
GAPDH
86 kDa
36 kDa
1
0.47
1.49
0.55
0.65
1.47
0.68
C
TGFβ+IL-1Ra+RNF182
TGFβ+IL-1Ra+RNF182+MFN2
Relative expression of mRNA
FN1
COL1A1
VIM
ACTA2
Fn1
Col1a1
Vim
Acta2

Figure S3

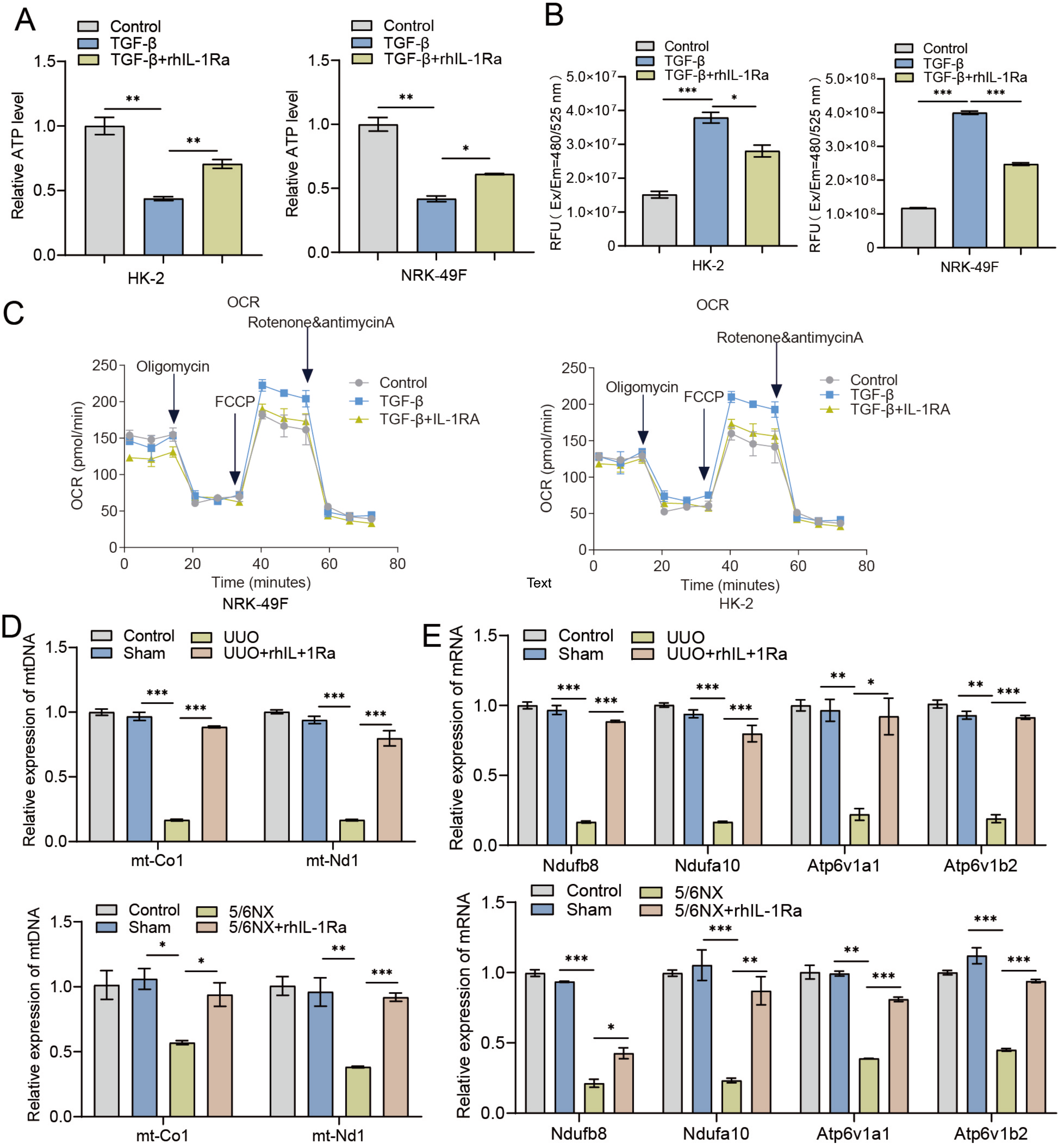
A
Control
TGF-β
TGF-β+rhIL-1Ra
Relative ATP level
HK-2
NRK-49F
B
RFU (Ex/Em=480/525 nm)
C
OCR
Oligomycin
FCCP
Rotenone&antimycinA
TGF-β+IL-1RA
OCR (pmol/min)
Time (minutes)
Text
D
Sham
UUO
UUO+rhIL+1Ra
Relative expression of mtDNA
mt-Co1
mt-Nd1
5/6NX
5/6NX+rhIL-1Ra
E
Relative expression of mRNA
Ndufb8
Ndufa10
Atp6v1a1
Atp6v1b2

Figure S4

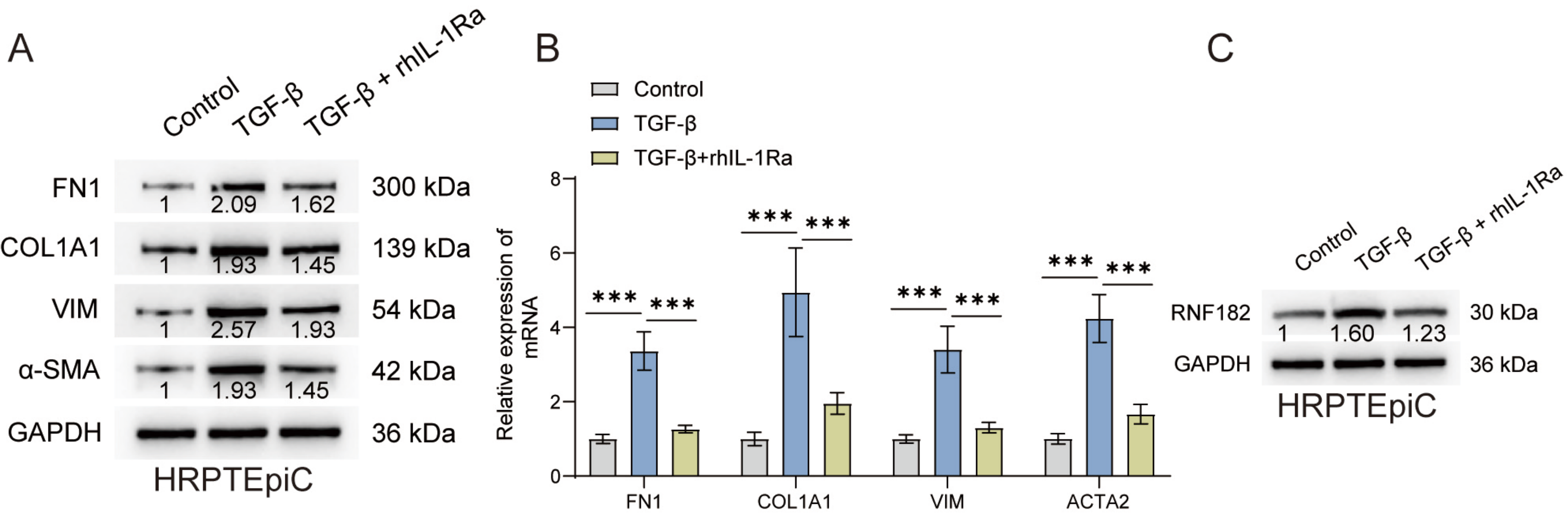
A
Control
TGF-β
TGF-β + rhIL-1Ra
FN1
1
2.09
1.62
300 kDa
COL1A1
1
1.93
1.45
139 kDa
VIM
1
2.57
1.93
54 kDa
α-SMA
1
1.93
1.45
42 kDa
GAPDH
36 kDa
HRPTEpiC
B
Control
TGF-β
TGF-β+rhIL-1Ra
Relative expression of mRNA
0
2
4
6
8
***
FN1
COL1A1
VIM
ACTA2
C
Control
TGF-β
TGF-β + rhIL-1Ra
RNF182
1
1.60
1.23
30 kDa
GAPDH
36 kDa
HRPTEpiC

Figure S5

Figure 1I

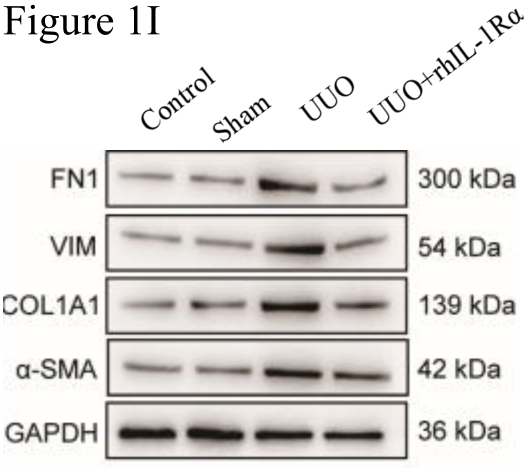

FN1

VIM

COL1A1

α-SMA

GAPDH

Figure 2H

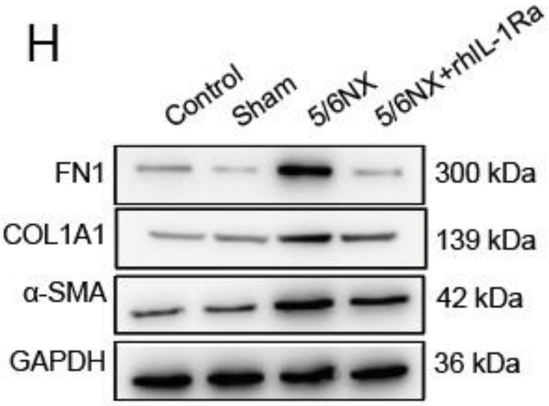

FN1

COL1A1

α-SMA

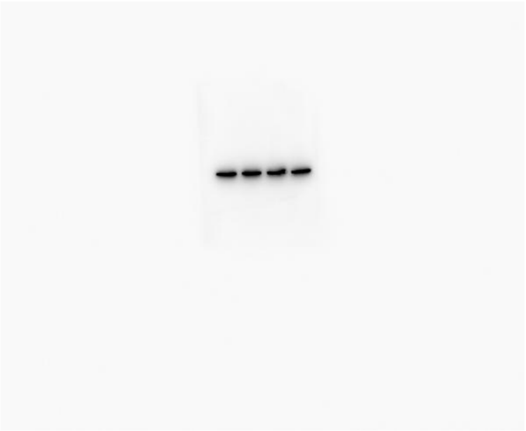

GAPDH

Figure 3C

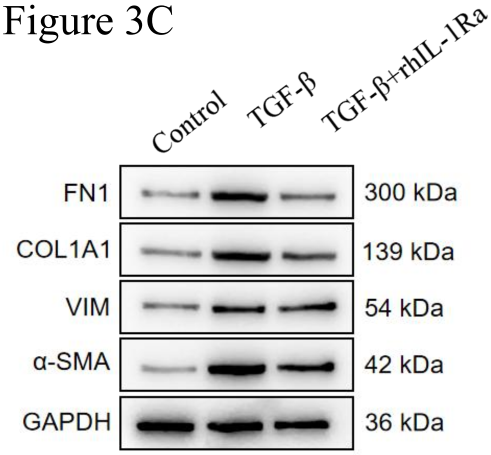

HK-2

FN1

COL1A1

VIM

α-SMA

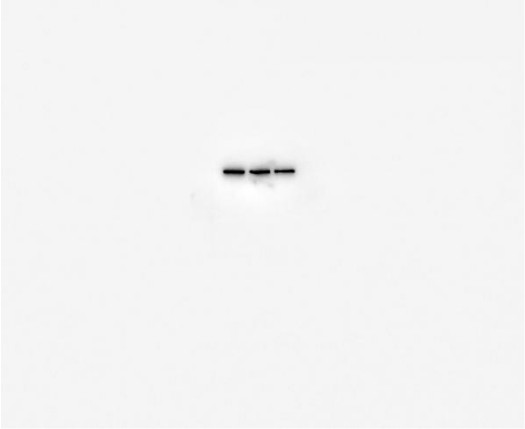

GAPDH

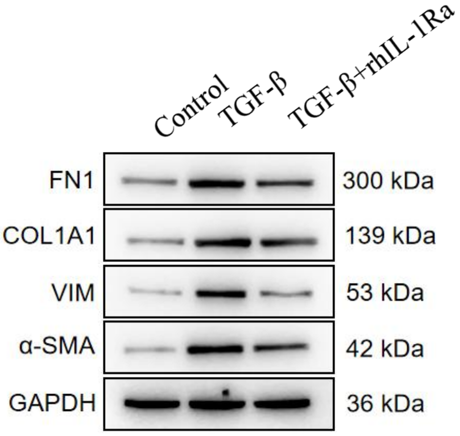

NRK-49F

FN1

COL1A1

VIM

α-SMA

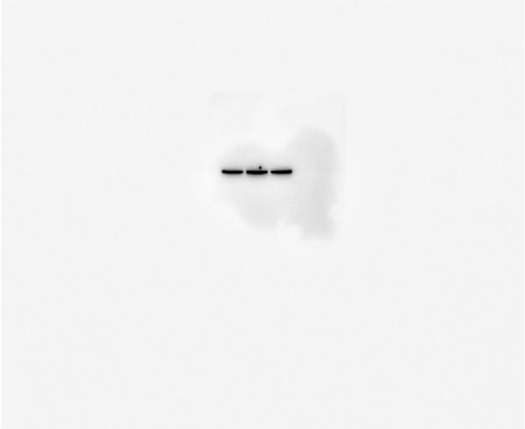

GAPDH

Figure 4C

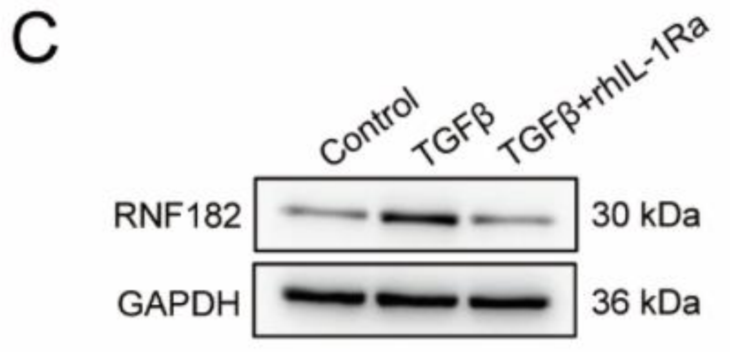

HK-2

RNF 182

GAPDH

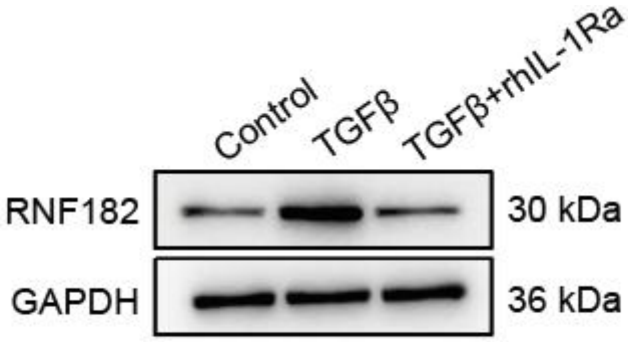

NRK-49F

RNF 182

GAPDH

Figure 4F

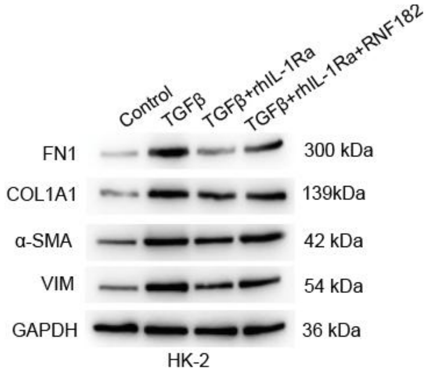

HK-2

FN1

COL1A1

α-SMA

VIM

GAPDH

NRK-49F

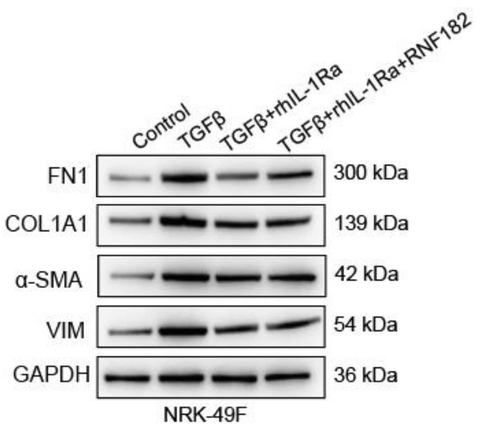

FN1

COL1A1

α-SMA

VIM

GAPDH

Figure 5B

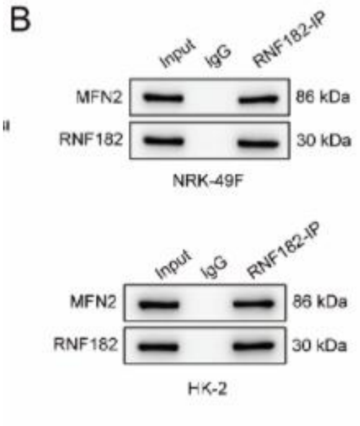

NRK-49F

MFN2

RNF 182

HK-2

MFN2

RNF 182

Figure 5C

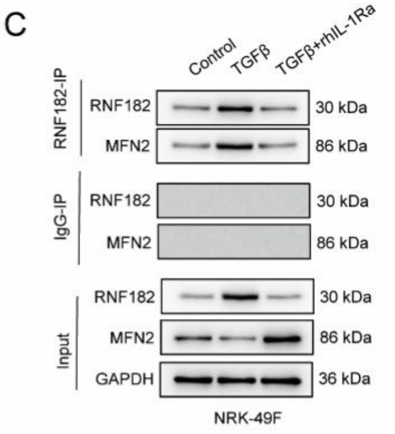

NRK-49F

RNF 182-IP

RNF 182

MFN2

Input

RNF 182

MFN2

GAPDH

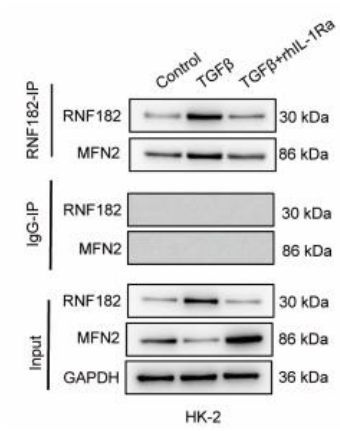

HK-2

RNF 182-IP

RNF 182

MFN2

Input

RNF 182

MFN2

GAPDH

Figure 5D

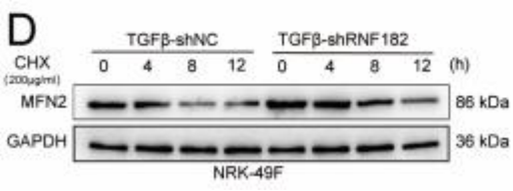

NRK-49F

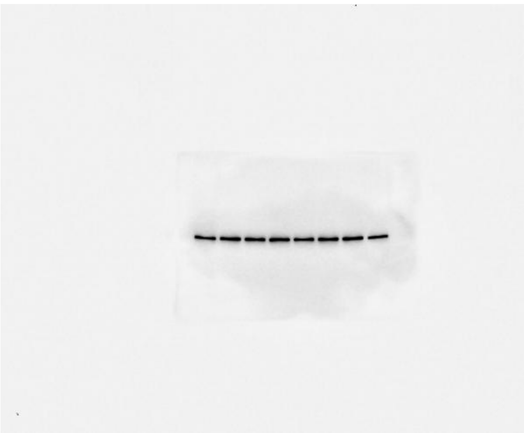

MFN2

GAPDH

HK-2

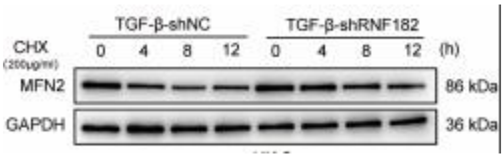

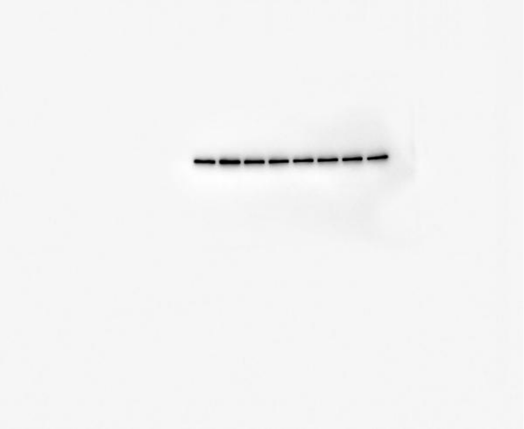

MFN2

GAPDH

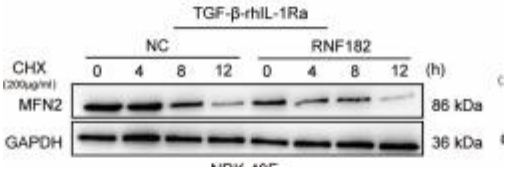

NRK-49F

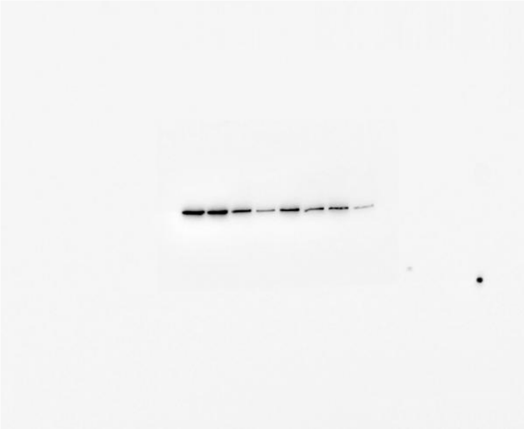

MFN2

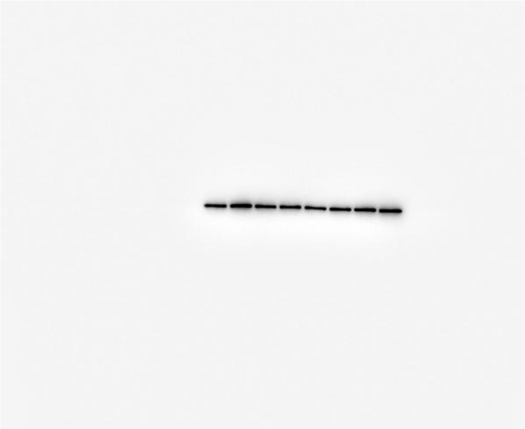

GAPDH

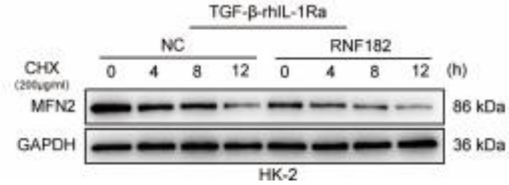

HK-2

MFN2

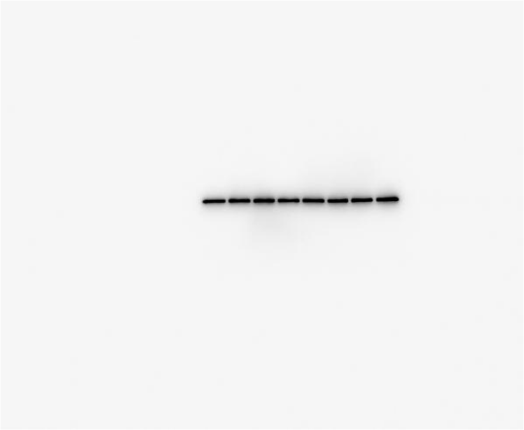

GAPDH

Figure 5E

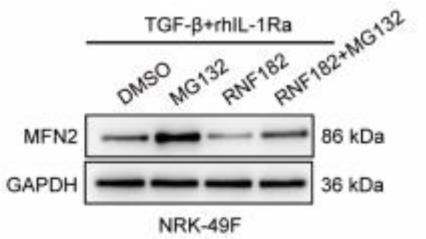

NRK-49F

MFN2

GAPDH

HK-2

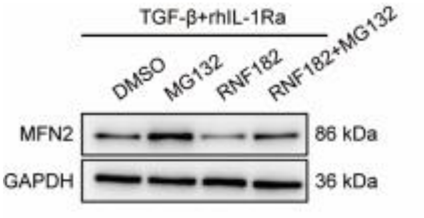

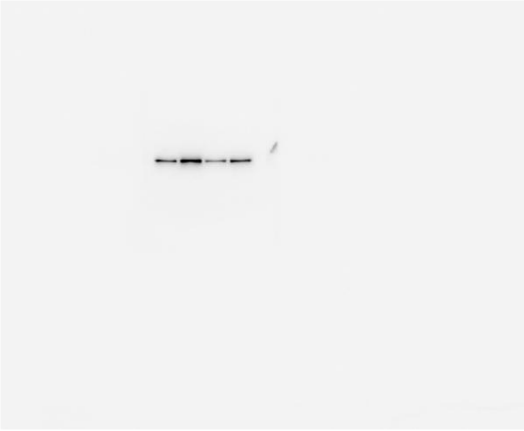

MFN2

GAPDH

Figure 5F

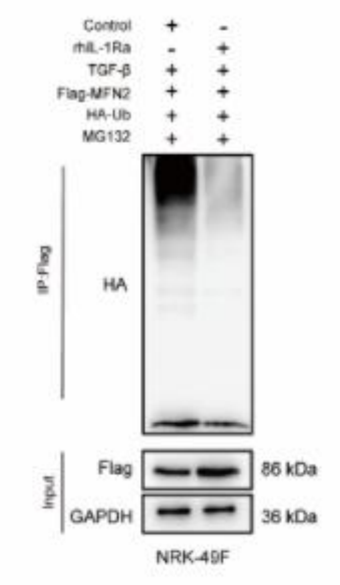

NRK-49F

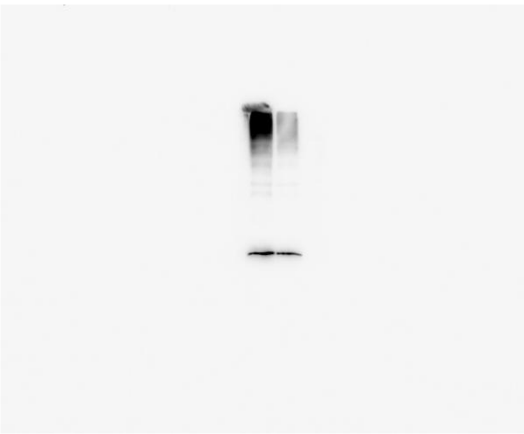

HA

INPUT

Flag

GAPDH

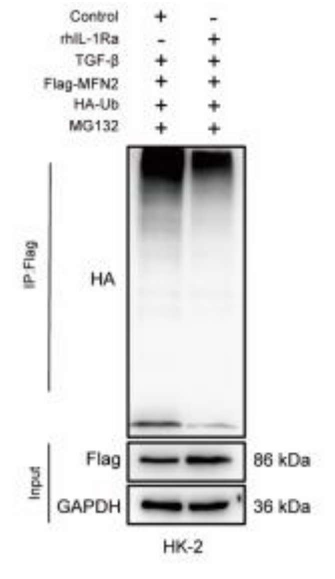

HK-2

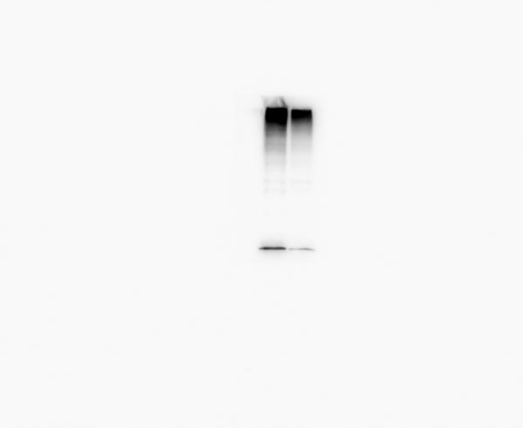

HA

Flag

GAPDH

Figure 5G

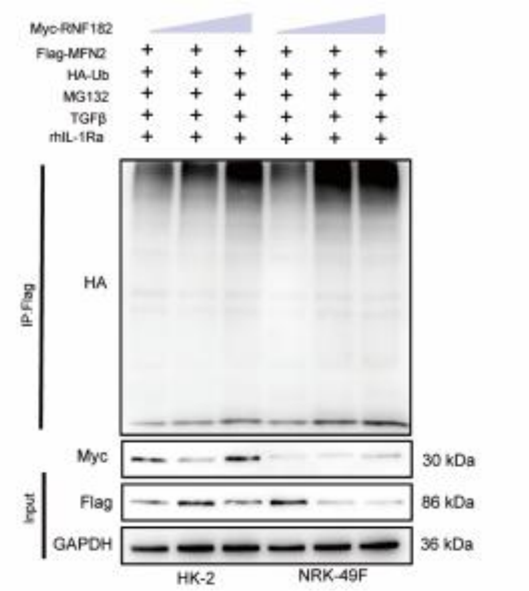

IP-Flag

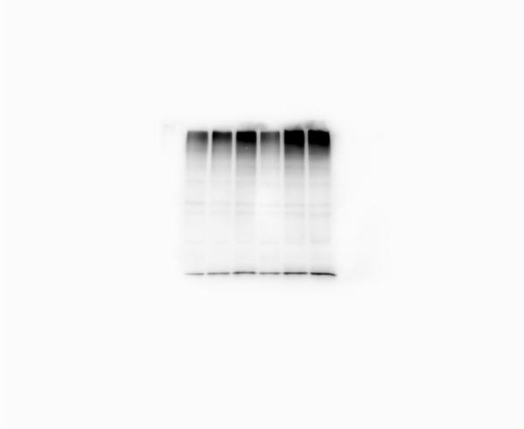

Input

Flag

Myc

GAPDH

Figure 6F

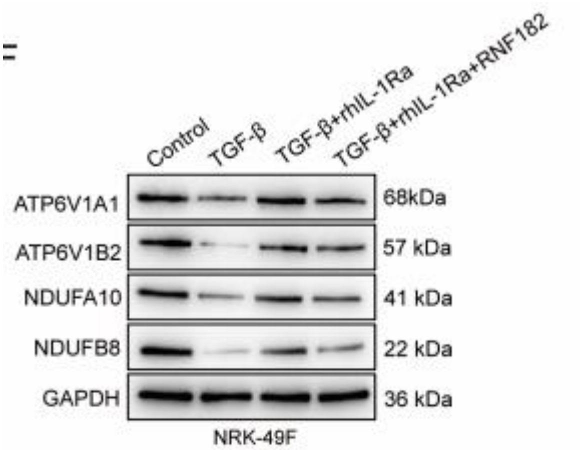

NRK-49F

ATP6V1A1

ATP6V1B2

NDUFA10

NDUFB8

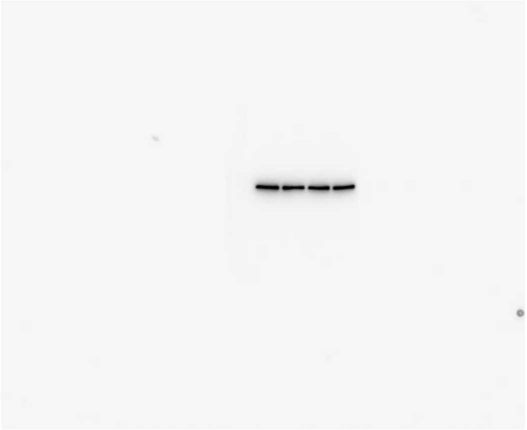

GAPDH

HK-2

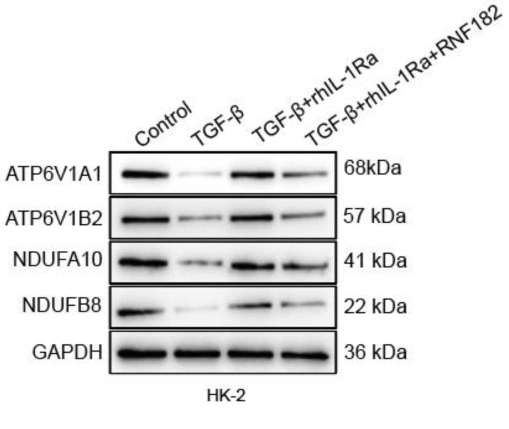

ATP6V1A1

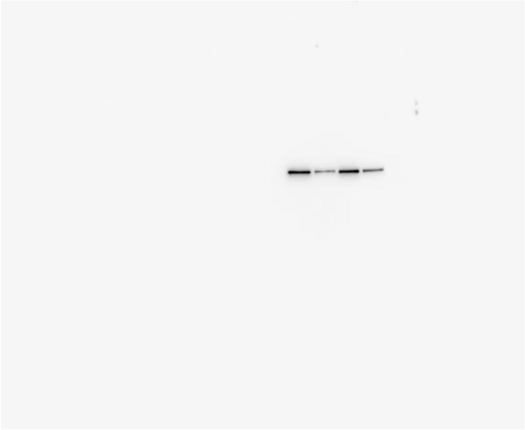

ATP6V1B2

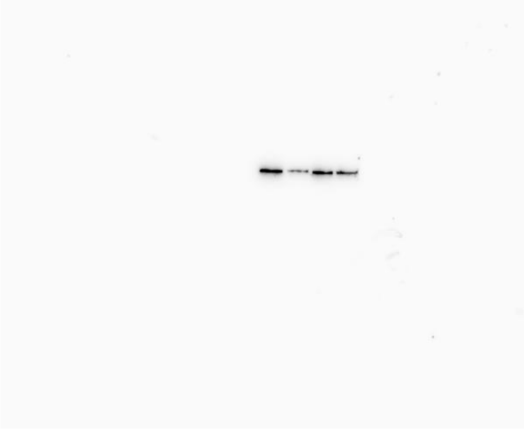

NDUFA10

NDUFB8

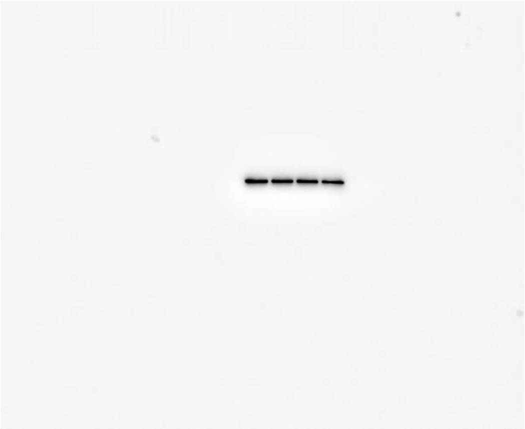

GAPDH

Figure S1C

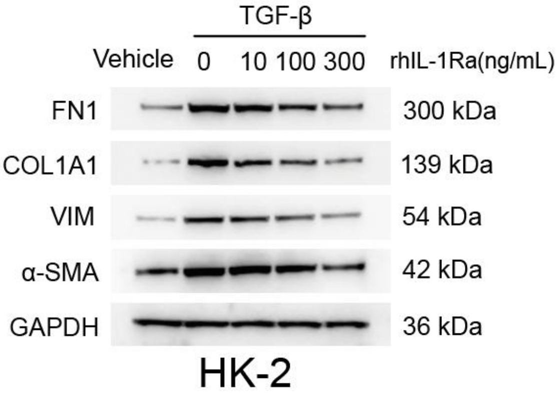

HK-2

FN1

COL1A1

VIM

α-SMA

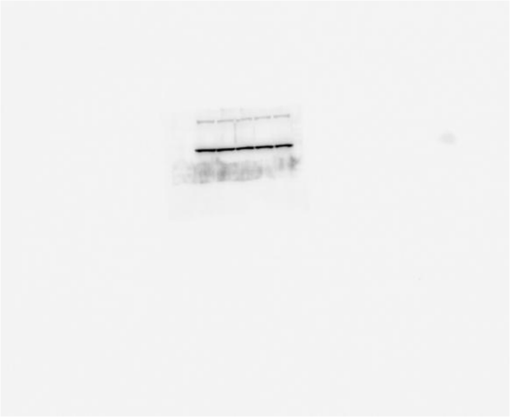

GAPDH

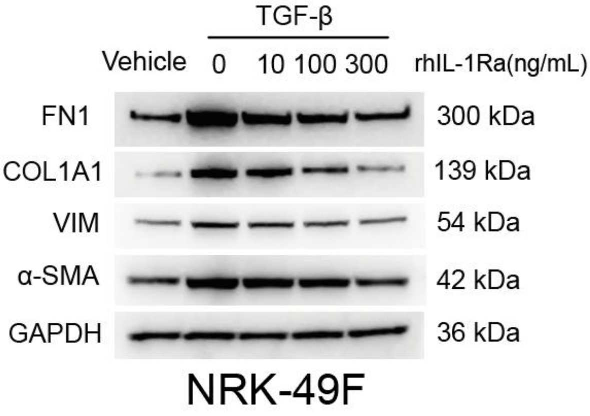

NRK-49F

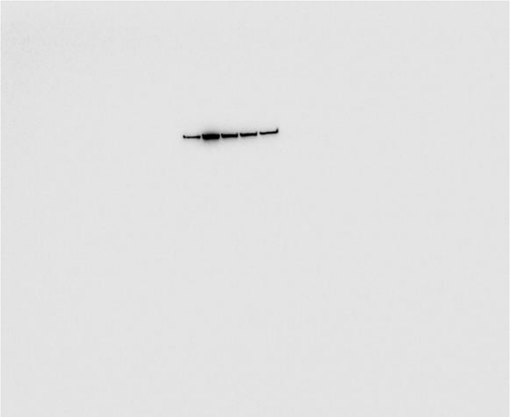

FN1

COL1A1

VIM

α-SMA

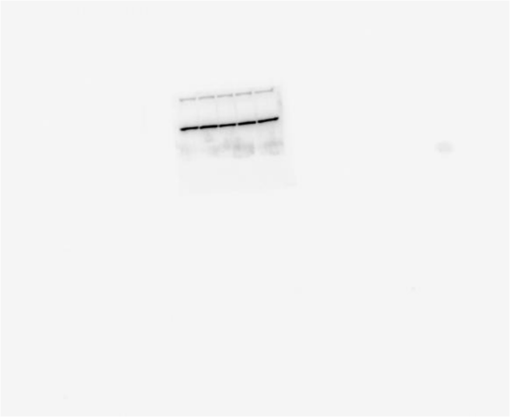

GAPDH

Figure S2C

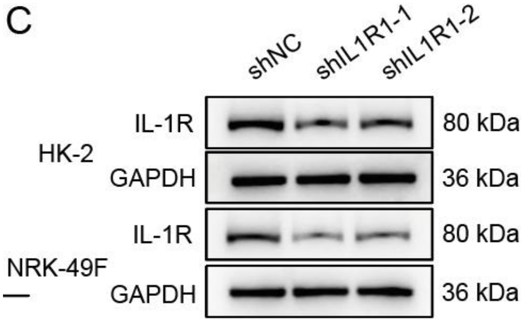

HK-2

IL-1R

GAPDH

NRK-49F

IL-1R

GAPDH

Figure S2E

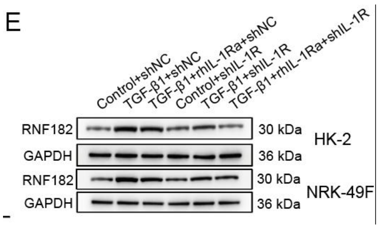

HK-2

RNF-182

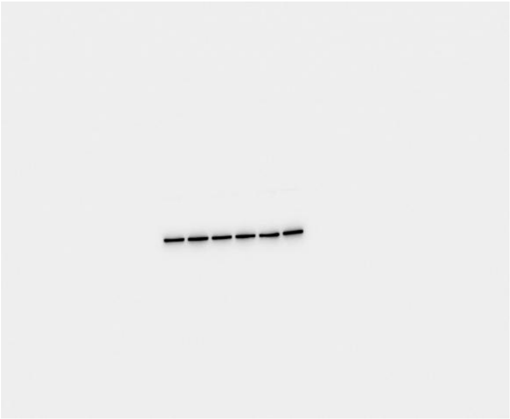

GAPDH

NRK-49F

RNF-182

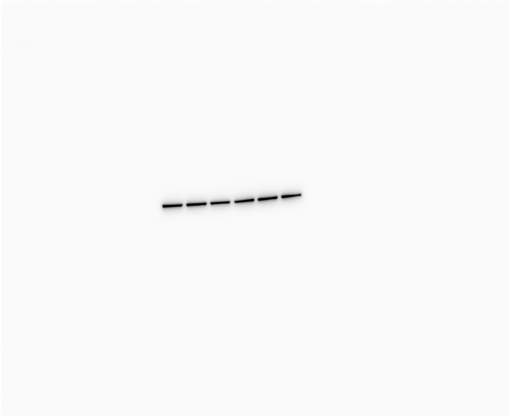

GAPDH

Figure S2G

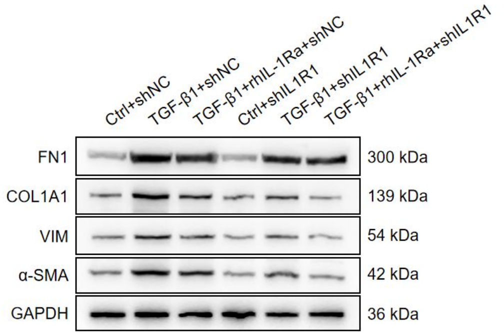

HK-2

FN1

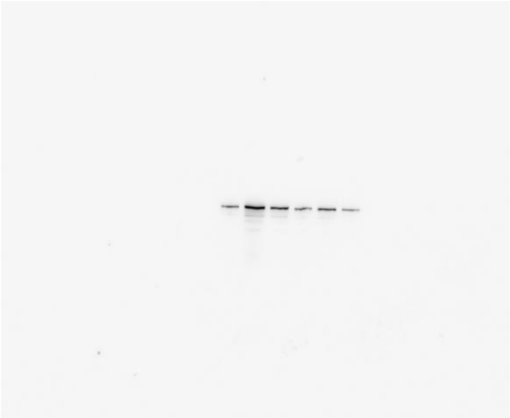

COL1A1

VIM

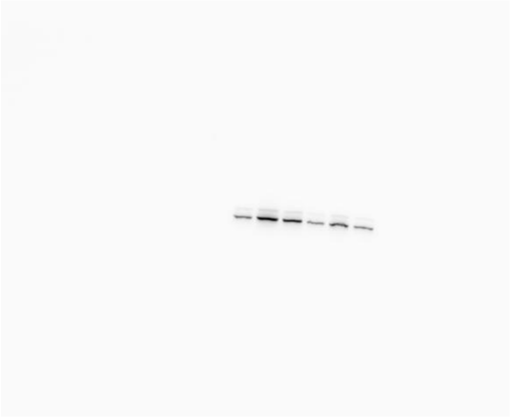

α-SMA

GAPDH

NRK-49F

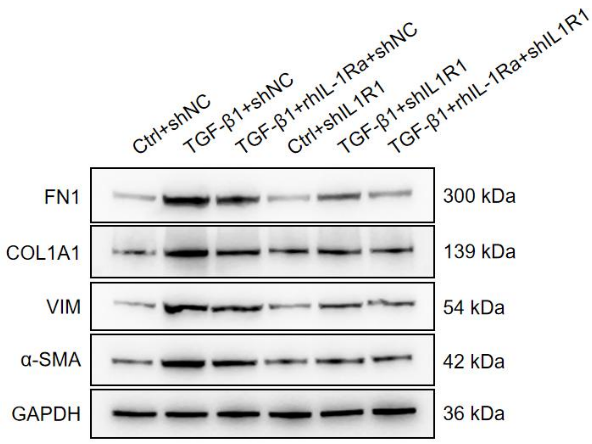

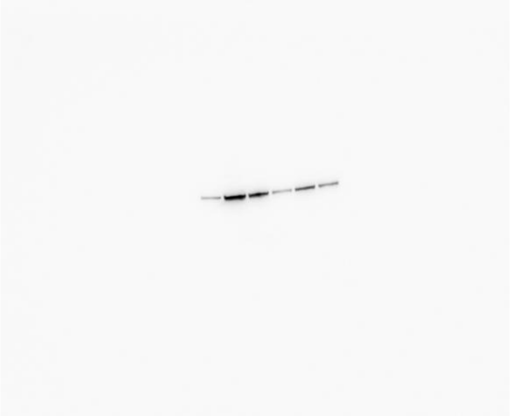

FN1

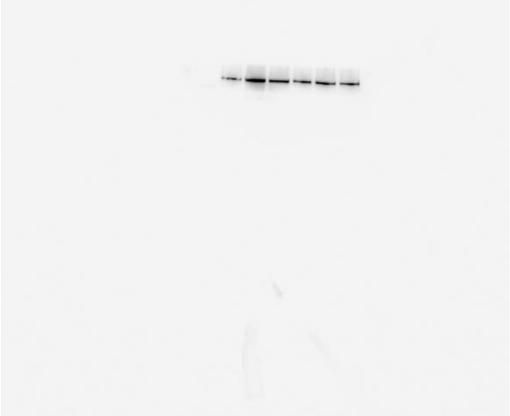

COL1A1

VIM

α-SMA

GAPDH

Figure S2I

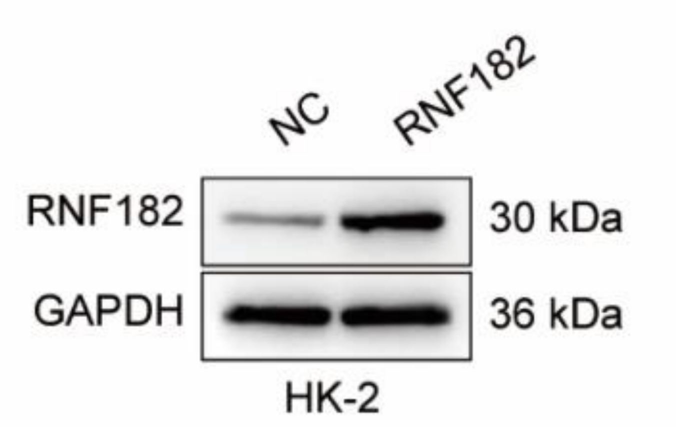

HK-2

RNF 182

GAPDH

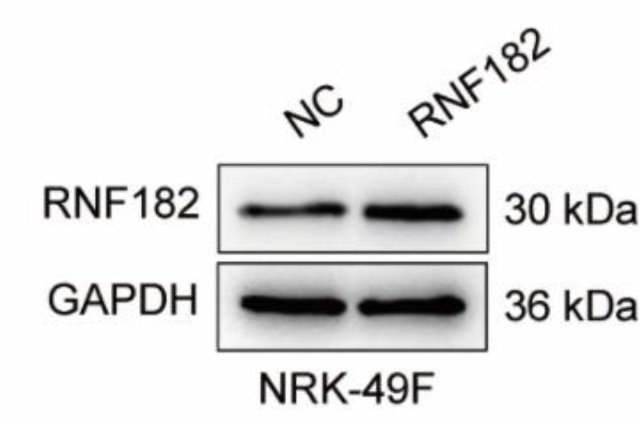

NRK-49F

RNF 182

GAPDH

Figure S3B

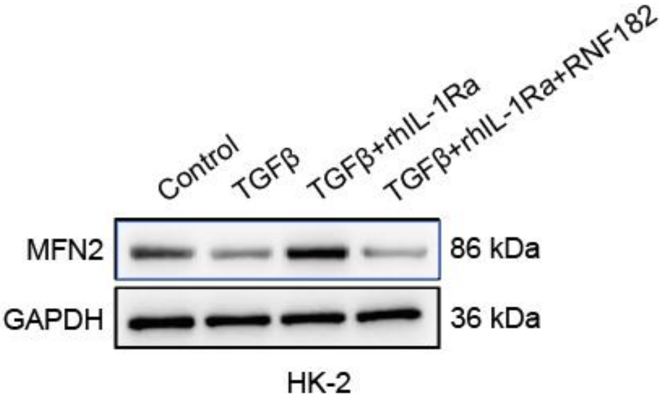

HK-2

MFN2

GAPDH

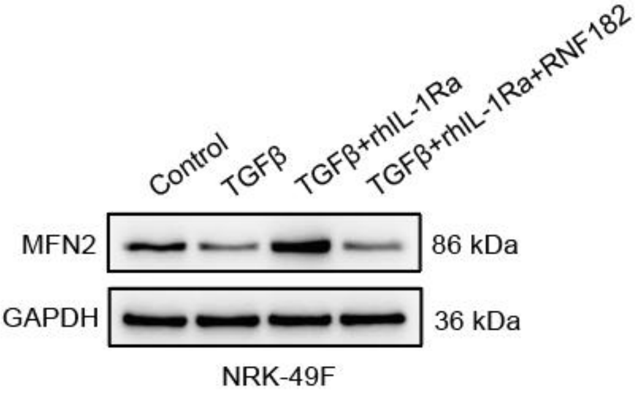

NRK-49F

MFN2

GAPDH

Figure S5A

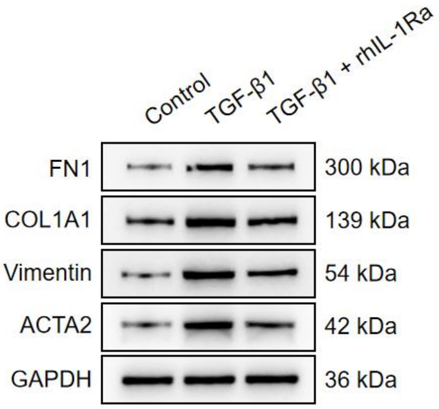

FN1

COL1A1

VIM

α-SMA

GAPDH

Figure S5C

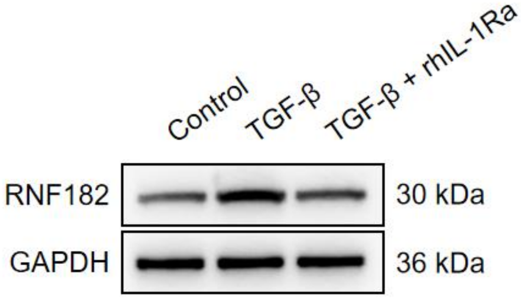

RNF182

GAPDH

Supplementary Figure Lengend

Figure S1.rhIL-1Ra demonstrates dose-dependent anti-fibrotic and anti-migratory effects in vitro. (A) Representative immunofluorescence images confirm the expression of Interleukin-1 Receptor (IL-1R, red) in both human kidney proximal tubular epithelial cells (HK-2) and rat kidney fibroblasts (NRK-49F). Nuclei were counterstained with DAPI (blue). (B) Dose-response analysis by qRT-PCR shows that rhIL-1Ra progressively suppressed the TGF-β1-induced mRNA expression of fibrotic markers (COL1A1, FN1, Vim, ACTA2) in both HK-2 (left) and NRK-49F (right) cells over a concentration range of 0–300 ng/mL. (C) This dose-dependent inhibition was confirmed at the protein level by Western blot, which showed reduced expression of FN1, COL1A1, VIM, and α-SMA in TGF-β1-stimulated cells. (D) Functionally, a wound healing assay demonstrates that rhIL-1Ra significantly inhibited the collective cell migration induced by TGF-β1 in both cell lines over 24 hours, with quantification of the migration rate shown in the bar graphs below. Scale bars = 100 μm. Data are presented as mean ± SEM. $^{**}p < 0.01$, $^{***}p < 0.001$.

Figure S2. IL-1R signaling is essential for TGF-β1-induced RNF182 expression and the subsequent pro-fibrotic response. Following qRT-PCR validation of RNA-seq candidates, which confirmed TGF-β1-induced upregulation of RNF182 is attenuated by rhIL-1Ra (A), the indispensable role of IL-1R was investigated. Efficient knockdown of IL-1R using shRNA, confirmed at both the mRNA (B) and protein (C) levels, completely abrogated the ability of TGF-β1 to induce RNF182 expression (D, E). Consequently, silencing IL-1R also prevented the TGF-β1-driven upregulation of the pro-fibrotic program, including key markers FN1, COL1A1, VIM, and ACTA2, at both the transcript (F) and protein (G) levels.

Additionally, the efficiency of the RNF182 overexpression vector used in subsequent experiments was confirmed at the mRNA (H) and protein (I) levels. Data are presented as mean ± SEM. Statistical significance was determined using one-way ANOVA with Tukey's post-hoc test or an unpaired two-tailed Student's t-test. ns: not significant, *$p < 0.05$, **$p < 0.01$, ***$p < 0.001$.

Figure S3. MFN2 expression is regulated post-transcriptionally, and MFN2 overexpression counteracts RNF182-driven pro-fibrotic gene expression. (A) Relative mRNA expression levels of MFN2/Mfn2 in HK-2 (left) and NRK-49F (right) cells under the indicated conditions, measured by qRT-PCR. Data normalized to GAPDH/Gapdh and shown relative to the Control group. (B) Representative Western blot analysis showing MFN2 protein levels in NRK-49F (top) and HK-2 (bottom) cells under the indicated conditions. Blots are representative of three independent experiments, and the values below the bands indicate the mean relative densitometric intensity (n=3) normalized to GAPDH. (C) Relative mRNA expression levels of fibrotic marker genes (*FN1/Fn1*, *VIM/Vim*, *COL1A1/Col1a1*, *ACTA2/Acta2* [encoding α-SMA]) in HK-2 (left) and NRK-49F (right) cells treated with TGF-β1+rhIL-1Ra and co-transfected with an RNF182 overexpression vector, either alone or together with an MFN2 overexpression vector, measured by qRT-PCR. Data normalized to *GAPDH/Gapdh* and shown relative to the group transfected with RNF182 alone. Data are presented as mean ± SEM. Statistical significance in (A) was determined using One-way ANOVA followed by Tukey's multiple comparison post-hoc test. Statistical significance in (C) was determined using an unpaired two-tailed Student's t-test comparing the two indicated groups. ns: not significant, *$p < 0.05$, **$p < 0.01$, ***$p < 0.001$.

Figure S4. rhIL-1Ra treatment preserves mitochondrial function and gene expression in vitro and in vivo.

(A) Relative intracellular ATP levels in HK-2 (left) and NRK-49F (right) cells treated with vehicle (Control), TGF-β1, or TGF-β1 plus rhIL-1Ra, measured using a luminescence-based ATP assay. Data normalized to the Control group. (B) ROS levels measured fluorescence (RFU, Relative Fluorescence Units, Ex/Em=480/525 nm) in HK-2 (left) and NRK-49F (right) cells treated with Control, TGF-β1, or TGF-β1+rhIL-1Ra. (C) Real-time OCR profiles from Seahorse XF Mitochondrial Stress Test in HK-2 (left) and NRK-49F (right) cells treated with Control, TGF-β1, or TGF-β1+rhIL-1Ra. Arrows indicate sequential injections of oligomycin, FCCP, and rotenone/antimycin A. (D) Relative mRNA expression levels of mitochondrial DNA-encoded genes (*mt-Co1, mt-Nd1*) in kidney tissues from mice subjected to UUO (top) or 5/6Nx (bottom) models, comparing Control, Sham, injury model, and injury model + rhIL-1Ra treatment groups, measured by qRT-PCR. Data normalized to Gapdh and shown relative to the Sham group. (E) Relative mRNA expression levels of *Ndufb8, Ndufa10, Atp6v1a1* and *Atp6v1b2* in kidney tissues from UUO (top) or 5/6Nx (bottom) models under the same conditions as in (D), measured by qRT-PCR. Data normalized to Gapdh and shown relative to the Sham group. Statistical significance was determined using One-way ANOVA followed by Tukey's multiple comparison post-hoc test. $^*p < 0.05$, $^{**}p < 0.01$, $^{***}p < 0.001$.

Figure S5. The anti-fibrotic mechanism of rhIL-1Ra via the RNF182/MFN2 axis is conserved in primary human kidney cells. Experiments were performed using primary Human Renal Proximal Tubular Epithelial Cells (HRPTEpiC). (A, B) rhIL-1Ra effectively counteracted the pro-fibrotic response induced by TGF-β1. Western blot (A) and qRT-PCR (B) analyses

demonstrate that rhIL-1Ra significantly attenuated the TGF-β1-induced upregulation of fibrotic markers (FN1, COL1A1, VIM, α-SMA). (C) Western blot analysis shows that rhIL-1Ra suppressed the TGF-β1-induced upregulation of the E3 ubiquitin ligase RNF182. GAPDH was used as a loading control. Data are presented as mean ± SEM. ***$p < 0.001$.

Supplementary Table 1

Gene	Species	Forward	Reverse
COL1A1	Human	ACAGAGGTTTCAGTGGTTTG	ACAGAGGTTTCAGTGGTTTG
FN1	Human	AGGAAGCCGAGGTTTTAACTG	AGGACGCTCATAAGTGTCACC
ACTA2	Human	CCTCATGAAGATCCTGACTG	ACAGTTTCTCCTTGATGTCC
RNF182	Human	TAGCCTGCCCGATGACAAC	CAGCAGCTCAGTAGGGTTCT
MFN2	Human	CACATGGAGCGTTGTACCAG	TTGAGCACCTCCTTAGCAGAC
COL3A1	Human	TAAGAATGGTGCCAAAGGAG	GATCCATCCTTGCCATCTTC
VIM	Human	GCTGCGAGAGAAATTGCAGGA	CCACTTTCCGTTCAAGGTCAAG
COL4A1	Human	CCAGGGGTCGGAGAGAAAG	GGTCCTGTGCCTATAACAATTCC
NDUFA10	Human	AAGCAATGATGGCAACAGTTACC	GCTCCAACACAACACCTTGTC
NDUFB10	Human	AAGCAATGATGGCAACAGTTACC	GCTCCAACACAACACCTTGTC
ATP6V1B2	Human	GCCTACCAGGACCGCTATCA	GTCATACAGAGCCACACAGCA
ATP6V1A1	Human	CAGCCTCGCCTCACATACAA	GTGCCATCCGGTAAGGTCAA
mt-CO1	Human	GGCCTGTACGTCAAGTTCGA	AGCAGTAGTTCCTCCCTGGA
mt-ND1	Human	GCCCACTTCCACTATGTCCT	TGGCGTAGGTTTGGTCTAGG
GAPDH	Human	ACCCTCACCACTACAATCTTCC	ATGAGTTGGTCGTAGCGGAA
Fn1	Rat	GGAGCCTTCACACATCACCA	GTGGCCTGGAATGGTAGCTT
Col1a1	Rat	GAGACAGGCGAACAAGGTGA	GGGAGACCGTTGAGTCCATC
Acta2	Rat	TGACTGAGCGTGGCTATTCC	CCATCAGGCAGTTCGTAGCT
Col3a1	Rat	AAGGCTGCAAGATGGATGCT	AGCTGAACTGAAAGCCACCA
Vim	Rat	TTCTCTGGCACGTCTTGACC	TCATACTGCTGGCGGACATC
Col4a1	Rat	CCTTGTGACCAGGCATAGTC	CGCTCGTTTCCTTGGACATA
Rnf182	Rat	TAGCCTGCCCGATGACAAC	CAGCAGCTCAGTAGGGTTCT
Ndufa10	Rat	AAGCAATGATGGCAACAGTTACC	GCTCCAACACAACACCTTGTC
Atp6v1b2	Rat	CCAGCCTCGTCTCACCTACA	CGGCTTTGGAGCCACTAACT
Atp6v1a1	Rat	CCGCCCAGAGTGACAATAAGA	GATCTTTGCCTCGCCATCCT
mt-Co1	Rat	AGGCTTCGGGAACTGACTTG	GGCTAGGTTTCCGGCTAAGG
mt-Nd1	Rat	GCAGGACCATTCGCCCTATT	AAAACGGGGGTAGGATGCTC
Gapdh	Rat	CAAAAGGGTCATCATCTCC	CTGACAATCTTGAGTGAGTT

Supplementary Table 2

WB-Anti-	Brand	Art.No.(article number)	Dilution
Collagen I	Abcam	Cat# ab34710	1:1000
Fibronectin 1 (FN1)	Abcam	Cat# ab2413	1:1000
α-Smooth Muscle Actin (α-SMA)	Sigma-Aldrich	Cat# A2547	1:2000
RNF182	Abcam	Cat# 72533	1:1000
Vimentin	CST	Cat# 5741	1:1000
MFN2	Abcam	Cat# ab56889	1:1000
Ubiquitin	CST	Cat# 3936	1:1000
Flag-tag	Sigma-Aldrich	Cat# F1804	1:2000
Myc-tag	CST	Cat# 2276	1:1000
HA-tag	CST	Cat# 3724	1:1000
NDUFB8	Abcam	ab110242	1:1000
SDHB	Abcam	ab14714	1:1000
UQCRC2	Abcam	ab14745	1:1000
MTCO1	Abcam	ab14705	1:1000
ATP5A	Abcam	ab14748	1:1000
GAPDH	CST	Cat# 5174	1:2000
